# A Cu(ii)-MOF based on a propargyl carbamate-functionalized isophthalate ligand†

**DOI:** 10.1039/d1ra02686k

**Published:** 2021-06-07

**Authors:** Maria Cristina Cassani, Francesca Gambassi, Barbara Ballarin, Daniele Nanni, Ilaria Ragazzini, Davide Barreca, Chiara Maccato, Antonietta Guagliardi, Norberto Masciocchi, Alessandro Kovtun, Katia Rubini, Elisa Boanini

**Affiliations:** Dept. of Industrial Chemistry “Toso Montanari”, Bologna University Viale Risorgimento 4 I-40136 Bologna Italy maria.cassani@unibo.it daniele.nanni@unibo.it +39 051 2093700 +39 051 2093623; CNR-ICMATE, INSTM, Dept. of Chemical Sciences, Padova University Via Marzolo 1 I-35131 Padova Italy; Dept. of Chemical Sciences, Padova University, INSTM Via Marzolo 1 I-35131 Padova Italy; Institute of Crystallography, To.Sca.Lab, National Research Council via Valleggio 11 I-22100 Como Italy; Dept. of Science and High Technology, To.Sca.Lab., University of Insubria via Valleggio 11 I-22100 Como Italy; Institute of Organic Synthesis and Photoreactivity, (CNR-ISOF) Via P. Gobetti 101 I-40129 Bologna Italy; Dept. of Chemistry “Giacomo Ciamician”, Bologna University Via Selmi 2 I-40126 Bologna Italy

## Abstract

A copper-based metal–organic framework (MOF) was prepared using a new linker, a 5-substituted isophthalic acid bearing a propargyl carbamate group, intended to provide a terminal alkyne function protruding from the material surface to generate supported gold species for potential catalytic applications. The novel material was fully characterized by spectroscopic analyses of different kinds: FTIR, Raman, EDX, and XPS, as well as by thermal and surface area measurements. Synchrotron X-ray diffraction data analysis, in particular, revealed that this MOF, labelled [Cu(1,3-YBDC)]·*x*H_2_O (*x* ∼ 2), where Y stands for the pendant alkYne and BDC for benzene dicarboxylate, contains a complex network of 5-substituted isophthalate anions bound to Cu(ii) centers, arranged in pairs within paddlewheel (or “Chinese lantern”) fragments of Cu_2_(μ-COO)_4_(D)_2_ formulation (D being a neutral Lewis base), with a short Cu⋯Cu distance of 2.633(4) Å. Quite unexpectedly, the apical atom in the paddlewheel structure belongs to the carbamate carbonyl oxygen atom. Such extra coordination by the propargyl carbamate groups drastically reduces the MOF porosity, a feature that was also confirmed by BET measurements. However, the MOF functionality is retained at the external crystal surface where 2% of active terminal alkynes is located.

## Abstract 2

A copper-based metal–organic framework with a novel 5-substituted isophthalic linker bearing a propargyl carbamate group protruding from the material surface.

## Graphical abstract

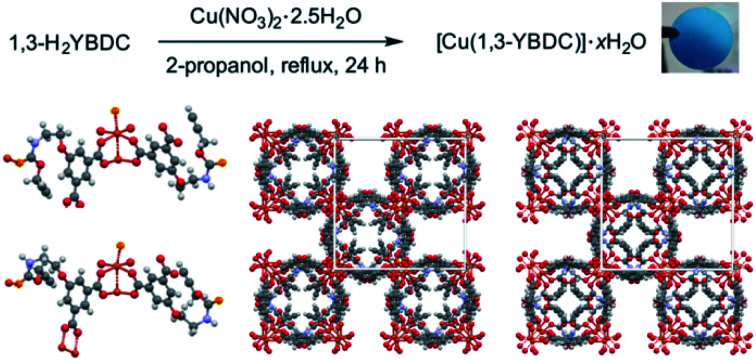

## 1. Introduction

Metal–Organic Frameworks (MOFs), also known as Porous Coordination Polymers (PCPs), are one of the most studied classes of crystalline solids, with a wide range of potential applications as functional materials in different advanced technological fields.^1–6^ The great effort made worldwide by scientists in academy and in industrial environments has provided, in the last two decades, a wealth of information on the viability of different synthetic methods (including post-synthetic modification approaches), on the stereochemical rules driving the formation of preferential topologies, and on the size or shape-selective aspects which depend on pore sizes and decoration, up to the very recent carbon capture industrial plants.^7^ Most of the MOF syntheses typically involve mixing metal salts (or pre-formed complexes), precipitated from organic solutions through reaction with a pre-formed organic linker. As denticity and other stereochemical preferences make the different organic ligands prone to the formation of archetypal frameworks (classified by their topology, or, more frequently, by labels referring to some of the most “popular”, or efficient, materials), it should not surprise that specific classes of ligands have received much more attention than others: benzene dicarboxylates (in the form of terephthalates and isophthalates), imidazolates, bis-pyrazolates, and topologically analogues with extended cores.^8^ Thus, a rational design of the network topology, with predictable size and shapes of the pores, has reached its maturity. Jointly with structural prediction, exemplified by the isoreticular chemistry approach, also thermal inertness, chemical stability in harsh conditions and, to some extent, the functional properties of the prepared MOFs can be rationally outlooked.^9–11^ However, when flexible residues are protruding from the ligand cores, the availability of a large “empty space” within the crystal makes prediction capability fail. In these conditions, a systematic study of the sample properties, which heavily depend on the actual “stable” structure, is required.

As anticipated, benzene-polycarboxylato ligands are frequent choices for the construction of stable and performing metal–organic networks. One of the most common organic linkers is benzene-1,3,5-tricarboxylic acid (BTC). The related 3D-[Cu_3_(BTC)_2_(H_2_O)_3_] compound (also called HKUST-1 or Cu–BTC),^12^ one of the most extensively studied 3-dimensional porous coordination polymers, is a highly porous material (with a specific surface area, SSA, reported to be *ca.* 690 m^2^ g^−1^ in the original paper^12^ and much higher than 1500 m^2^ g^−1^ in the marketed materials) in which {Cu_2_} units are coordinated to four carboxylate groups to give the well-known paddle-wheel unit.^6,13–17^ Simpler ligands, like benzene-dicarboxylates (BDC), thanks to their structural rigidity (and simultaneous charge balancing effects of M(ii) centers), have also been widely employed, as they can bear an additional, pendant and chemically tailored, functionality on the benzene ring.^18–20^

In recent years, our research group has shown that a propargyl carbamate [–N(H)C(O)O–CH_2_–C≡CH] group anchored on different oxide supports (SiO_2_, Al_2_O_3_, TiO_2_, Fe_3_O_4_) is capable of straightforwardly reducing Au(III) to Au(0), yielding supported gold nanoparticles (AuNPs) without the addition of any external reducing and/or stabilizing agent.^21–23^ The reactivity of the triple bond within this molecule has been additionally exploited to fabricate a novel amino-sulfide branched silica support by radical click thiol-yne (TYC) chemistry.^24^ Based on these results and taking into account that the chemical, structural, and functional behaviour of the gold nanoparticles reported above depend on the physico-chemical environment dictated by the support, we envisaged that the versatility demonstrated by the propargyl carbamate residue could be further exploited by anchoring it to different solid supports than oxides, for example within alkynyl-derivatized MOFs. Indeed, the isolation of a metal–organic framework starting from a suitably functionalized organic linker would ensure an even, dense, and possibly highly symmetric distribution of the reactive alkyne moiety in the material. Additionally, the material porosity and the presence of the metal nodes could affect the reactivity of the alkyne and induce different effects on the ensuing gold nanoparticles, than when the alkyne is attached onto the oxidic surfaces cited above.

In this paper we report the synthesis of a new organic linker belonging to the substituted BDC class, *i.e.* 5-(2-{[(prop-2-yn-1-yloxy)carbonyl]amino}ethoxy)isophthalic acid (which we have labelled 1,3-H_2_YBDC, where Y stands for alkYne and BDC for benzene dicarboxylate), bearing a propargyl carbamate residue; we also discuss the optimized conditions for the synthesis of the novel Cu(ii)–MOF, [Cu(1,3-YBDC)]·*x*H_2_O derived therefrom, and, finally, we present a detailed, comprehensive multi-technique characterization by means of complementary analytical, structural and imaging tools.

## 2. Results and discussion

### 2.1 Synthesis and characterization of [Cu(1,3-YBDC)]·*x*H_2_O

**Scheme 1:**
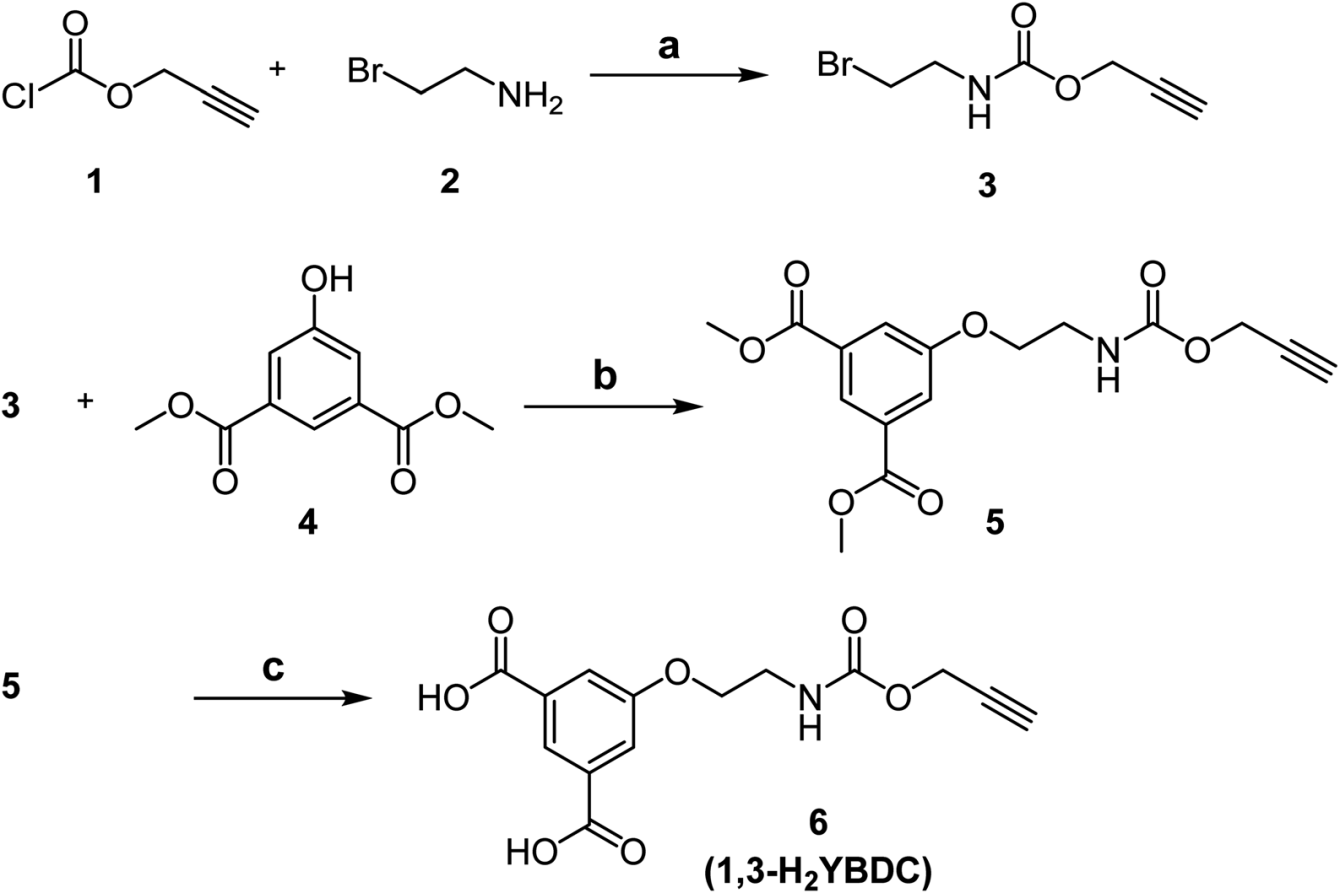
Synthesis of 6 (1,3-H_2_YBDC). Reagents and conditions: (a) NaHCO_3_ (3 eq.), THF/H_2_O, r.t., overnight; (b) NaI (1.5 eq.), Cs_2_CO_3_ (1.5 eq.), 2-butanone, 80 °C, overnight; (c) LiOH (2.0 M), THF/MeOH, r.t., 3 h.

The novel organic dicarboxylic linker 1,3-H_2_YBDC, bearing a propargyl carbamate group, was prepared following the synthetic route depicted in Scheme 1. The synthesis involves initial condensation of the commercially available chloroformate 1 with bromoamine 2, yielding carbamate 3. Crude 3 was then reacted with phenol 4 in the presence of NaI and cesium carbonate to afford arylether 5, which was subsequently converted into the corresponding dicarboxylic acid 6 by LiOH-promoted hydrolysis, with overall, reproducible yields normally well above 95%

**Scheme 2:**
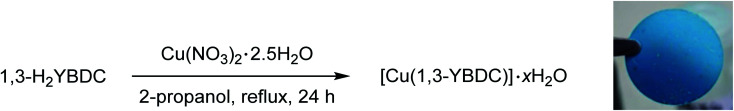
Synthesis of [Cu(1,3-YBDC)]·*x*H_2_O and image of a pellet made by pressing at 100 bar for 2 min its polycrystals.

As shown in Scheme 2, the final MOF was obtained by reacting the dicarboxylic species 1,3-H_2_YBDC with Cu(NO_3_)_2_ · 2.5H_2_O in refluxing 2-propanol for 24 h employing a Cu : L molar ratio of 1.8 : 1 (see Experimental section for further details).

Employment of 2-propanol was found crucial: indeed the use of ethanol brought about partial esterification of the linker, hence causing erratic MOF yields and purity.^6^ After filtration, a turquoise polycrystalline powder was obtained with yields above 90%. Thanks to the nearly quantitative formation of compound 6, the overall yield of the entire process approaches 90%, making the scale-up of this efficient synthesis highly viable.

X-ray evidences, shown in the ESI† file and commented in the Experimental section, highlight the formation of Cu(OH)_2_ and Cu_2_(OH)_3_NO_3_, in variable (minimal, but visible) amounts. Even by keeping them at a minimum, still a few percent (w/w%, from TGA data) of these hydroxy-salts are always present and contaminate the MOF up to a maximum value of *ca.* 10 w/w%. X-ray powder diffraction, the most suitable technique for assessing the correct phase composition of the mixture, however, did not help in quantifying it, as the extreme texture of the contaminants made visible only a few (00ℓ) diffraction peaks. Indeed, crystal phase quantification by the Rietveld method^25^ relies on the experimentally determined scale factors and, if preferential orientation (texture) is present, on the accurate estimation of the so-called orientation distribution function.^26^ In the present case, where (00ℓ) diffraction peaks only were observed, neither the ODF nor the scale factor of the fully oriented (nanosized) crystal plates are accessible, as they mutually 100% correlate.

Finally, the target product is stable in water at r.t and at neutral pH and such stability was evaluated using XRD. After keeping the product in water for 24 h, no changes in the XRD pattern were observed (see the ESI file†).

### 2.2 Vibrational spectroscopy and thermal characterization

The coordination of the Cu^2+^ ions to the organic linkers in [Cu(1,3-YBDC)]·*x*H_2_O was first analysed by IR and Raman spectroscopy. As shown in the ESI file,† in the 2800–3200 cm^−1^ region of the ATR-IR spectrum of the Cu–MOF the very broad band due to the carboxylic acid OH stretching present in the ligand is totally absent, indicating that full deprotonation occurred, with the carboxylate ions involved in binding to the metal centres, very much as in similar compounds.^27^

In addition, the IR spectrum (Fig. S13†) possesses two intense bands at 1585 and 1374 cm^−1^ which are particularly diagnostic for the asymmetric and symmetric stretching mode of the carboxylate group (RCO_2_^−^). The difference Δ*ν* (213 cm^−1^) between the *ν*_as_(1585 cm^−1^) and *ν*_s_(1372 cm^−1^) stretching vibrations indicates that each carboxylate group is bonded to two different copper atoms in a bridging bidentate mode, whereas the band at 731 cm^−1^ can be attributed to bonding between copper and oxygen of YBDC.^15,28–30^ Another band, attributed to the *ν*(C=O) stretching of the carbamate residue, bound to the Chinese lantern in apical position (see Crystallochemical analysis), can be found at 1628 cm^−1^, *i.e.* at *ca.* 59 cm^−1^ lower frequencies than in the pristine ligand. This red shift is in line with previous observations reported for analogous fragments coordinating, through their carbonylic oxygen atoms, to Cu(ii) ions of molecular (mononuclear) complexes.^31^

Complementary information was gained by Raman spectroscopy; Fig. S14 in the ESI† file compares the spectra measured for [Cu(1,3-YBDC)]·*x*H_2_O, 1,3-H_2_YBDC, and Cu(NO_3_)_2_ · 2.5H_2_O. The appearance of the Raman active band at 2135 cm^−1^, unobserved in the IR spectra, is assigned to –C≡C– stretching of the alkyne triple bond,^28^ and confirms that, during complexation, no changes in the structure of the organic linker have occurred nor is the triple bond involved in π–metal bond. The signals located at 1605 and 1004 cm^−1^ are associated with the benzene ring *ν*(C=C) stretching modes, whereas the peaks at 807 and 745 cm^−1^ are ascribed to out-of-plane *δ*(C–H) and *δ*(C=C) ring bending vibrations, respectively.^32,33^ In the low-frequencies region (600–200 cm^−1^ range), [Cu(1,3-YBDC)]·*x*H_2_O exhibits, in agreement with previous studies, two peaks at 495 and 276 cm^−1^, respectively assigned to the vibrational stretching modes of equatorial and axial Cu–O bonds.^34,35^

**Fig. 1:**
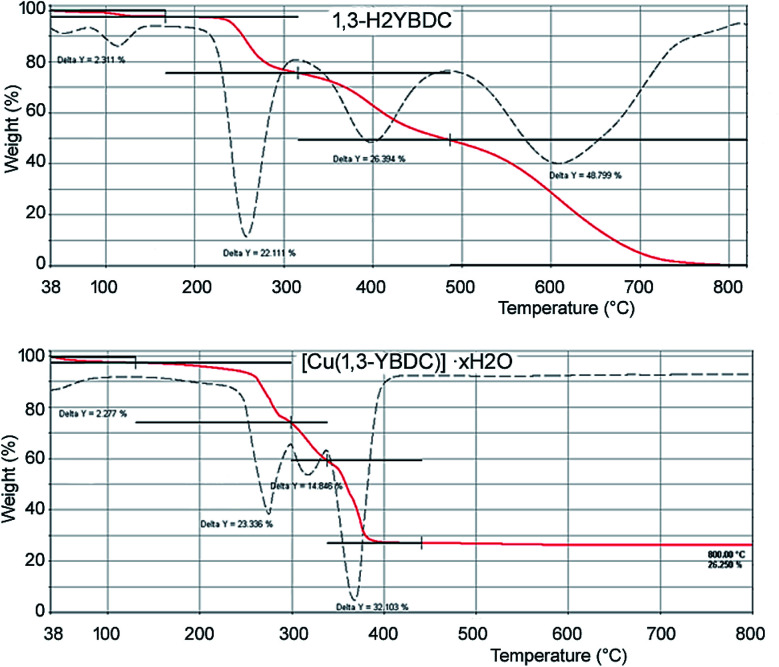
TGA curves (continuous red lines) and their first derivatives (dashed lines) for 1,3-H_2_YBDC (top) and [Cu(1,3-YBDC)]·*x*H_2_O (bottom).

The elemental analysis of the synthesized sample is in good agreement with the theoretical chemical composition, showing a Cu/N molar ratio of *ca.* 1 (see Experimental part). Thermogravimetric analyses (TGA) were carried out in air to determine the thermal stability of [Cu(1,3-YBDC)]·*x*H_2_O. The corresponding TGA profiles of 1,3-H_2_YBDC and [Cu(1,3-YBDC)]·*x*H_2_O are reported in Fig. 1. The two contaminants found in small quantities in the synchrotron X-ray diffraction analysis (*vide infra*) decompose at *ca.* 132 °C (*T*_onset_ for Cu(OH)_2_)^36^ and at *ca.* 175 °C (*T*_onset_ for Cu_2_(OH)_3_NO_3_)^37^ and are not evidenced in the TG trace, confirming their low percentage.

In the ideal absence of contaminants, TGA experiments can be safely used to estimate the amount of water content, but, as [Cu(1,3-YBDC)]·*x*H_2_O contains water molecules loosely bound to the framework (*vide infra*), very much as the prototypical channel hydrates found in zeolites and in molecular organic species of pharmaceutical interest, their amount can vary depending on the history of the sample and on the relative humidity conditions.^38^ Accordingly, the stoichiometry of this crystal phase is somewhat undefined, and may vary depending upon the external conditions.

The TGA plot for 1,3-H_2_YBDC (of C_14_H_13_NO_7_ formula, mw 307.25 g mol^−1^) shows three main weight losses between 200 and 600 °C, due to organic material decomposition, and zero residual weight. The first two steps are interpreted by progressive loss of the propargyl fragments (obs. 48.5%, calc. for C_6_H_8_NO_3_ 46.3%). Similarly, the copper-containing product (of C_14_H_11_CuNO_7_ formula, mw 368.79 g mol^−1^) shows two decomposition steps in the 200–350 °C range (attributed, as above, to the loss of the C_6_H_8_NO_3_ residue, obs. 38.2%, calc. 38.5%), terminating at a temperature *ca.* 150 °C lower than in the pristine organic ligand. Such lower thermal stability of the organic skeleton within the MOF is tentatively attributed to assistance, during decomposition, of redox process(es) catalyzed by Cu(ii) ions. Furthermore, a residue of ≈26 wt% is present at 400 °C, with no significant variation up to 800 °C, which is presumably due to residual CuO. As a residual 21.6% only is calculated if the starting material were pure, the excess residue at high *T* speaks for the presence of carbonaceous residuals and only marginally to Cu-rich contaminants, the nature of which is presented in the Experimental section.

### 2.3 Crystallochemical analysis

**Fig. 2:**
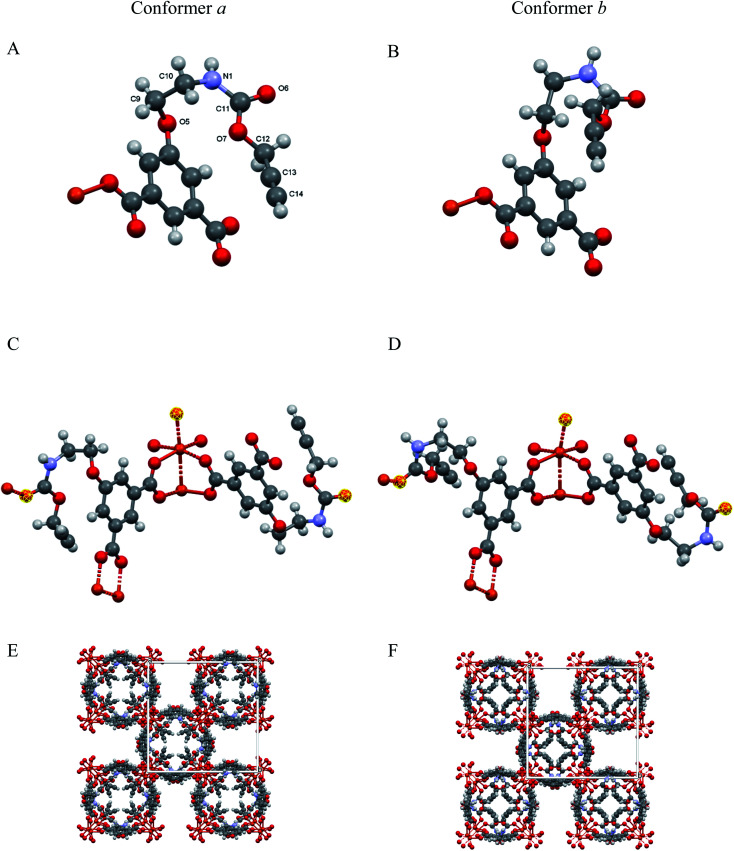
(A and B) Asymmetric unit showing the distinct conformations (conformers *a* and *b*) of the flexible propargyl carbamate residues (with labeling scheme in A); (C and D) The paddlewheel moiety and the location of the O6 atoms (highlighted in yellow) completing the Cu(ii) coordination through C=O⋯Cu bonds; (E and F) crystal packing, viewed down [001], for the two ordered and periodic models, highlighting extended channels, running in the c direction, and accounting, in both cases, for ≈20% of the crystal volume.

The solid [Cu(1,3-YBDC)]·*x*H_2_O (*x* ∼ 2) phase contains a complex network of 5-substituted isophthalate anions bound to Cu(ii) centers belonging to the common paddlewheel dimeric fragments (also known as “Chinese lantern”) of Cu_2_(μ-COO)_4_(*D*)_2_ formulation, D being a neutral Lewis base, typically bound through a N or O atom (see Fig. 2). The dimer is bisected by a twofold axis running along [110] of the *P*4/*ncc* space group and structurally similar to the archetypical Cu_2_(μ-acetate)_4_(H_2_O)_2_ molecule. In line with previous data for analogous compounds,^39^ it shows a short Cu⋯Cu distance of 2.633(4) Å [*vs.*, *e.g.*, 2.6107(4) Å in Cu_2_(μ-acetate)_4_(H_2_O)^40^ and 2.628(2) in HKUST-1 (ref. 12)] and similar carboxylate Cu–O bond distances [1.95–2.08 Å, avg. value 2.00 Å *vs.* 1.94–1.99 Å, avg. 1.97 Å for Cu_2_(μ-acetate)_4_(H_2_O)_2_ and 1.95 Å for HKUST-1 (ref. 12)]. Unexpectedly, the apical D atom in [Cu(1,3-YBDC)]·*x*H_2_O belongs to the carbonyl atom of the long propargyl carbamate residue, with Cu–O distances of 2.26 (O6a) and 2.16 Å (O6b), both with large e.s.d's (see Table 1). The propargyl carbamate residue is indeed disordered, with two alternative chain conformations of estimated 0.51(1):0.49(1) sof, and extra residual electron density in the crystal cavities. The two conformations, *a* and *b*, possess significantly different torsional angles, comparatively collected in Table 1, and illustrated in Fig. 2 (A and B panels). In order to simplify the understanding of the stereochemical features of this severely disordered crystal structure beyond the conventional cif file (containing the entire list of atoms in the asymmetric unit and of their site occupancy factors), we separately provide (in two additional files within the ESI†) the “cleaned” models for mutual comparison, referring to *a* and *b* conformers of the long propargyl carbamate residue. To further clarify the real situation, in our MOF with disordered residues, (half of) the crystal voids are filled by organic branches in two different conformations, within distinct channels which do not cross-talk one to the other, being several Å away. This means, in turn, that the crystal is aperiodic, and that the assumption of infinite crystal periodicity only addresses a statistical average of uncorrelated atomic positions (and movements), which is at the basis of Braggs' approach.

**Table 1:** Comparative conformational analysis of the *a* and *b* propargyl carbamate residues^a^

	Residue *a*	Residue *b*
Chain sof	0.51(1)	0.49(1)

**Torsion angles**		
C2–C3–O5–C9, °	26(1)	53(1)
C3–O5–C9–C10, °	93(1)	163(1)
O5–C9–C10–N1, °	35(3)	−108(3)
C9–C10–N1–C11, °	−104(4)	24(6)
C10–N1–C11–O7, °	18(7)	−48(8)
N1–C11–O7–C12, °	163(6)	−53(9)
C11–O7–C12–C13, °	156(9)	−162(10)

**Bond distance**		
Cu–O6, Å	2.26(10)	2.14(8)

a Labelling of atoms is shown in Fig. 2A.

The extended fragments shown in panels C and D highlight the location of the carbonyl oxygen atoms (O6a and O6b) completing the Cu_2_(μ-carboxylate)_4_ coordination sphere in apical positions. The complete structures (E and F panels) for the idealized and periodic *a* and *b* conformers (not including the weakly bound water atoms) show the terminal alkyne moieties pointing coherently into the same (tetrad axis-generated) crystal cavity. The remaining channels, accounting for ≈20% void space in both *a* and *b* models, are partially filled by water molecules, possibly disordered and of non-stoichiometric character. As powder diffraction of such complex material can provide only limited information on these guests, their amount and geometrical properties will not be discussed any further.

**Fig. 3:**
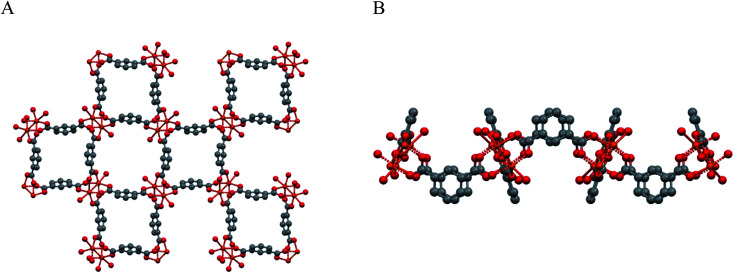
Sketches of the weakly corrugated 2D layers in the crystal packing of Cu_2_(μ-isophthalate)_4_D_2_, present also in [Cu(1,3-YBDC)]·*x*H_2_O. (A) View down [001]. (B) View down [100]. Note that, in [Cu(1,3-YBDC)]·*x*H_2_O, the layers are not separated, but are interlinked by the long propargyl carbamate residues (in both *a* and *b* conformations) completing the Cu_2_(μ-COO)_4_ coordination sphere (the apical D atom) with a truly 3D connectivity.

A simplified overall picture of the crystal structure is shown in Fig. 3 by removing the H atoms and the propargyl carbamate moiety from the crystal framework, but leaving the carbonylic oxygen atom bound to Cu. These sketches highlight the presence of separated (weakly corrugated) 2D layers, very similar to those found in [Cu(1,3-BDC) (C_5_H_5_N)], where D = pyridine N atom.^41^ However, in [Cu(1,3-YBDC)]·*x*H_2_O, the layers are not disjointed, but are interlinked by the long propargyl carbamate residues (in both *a* and *b* conformations) completing the Cu_2_(μ-COO)_4_ coordination sphere with the apical O6a and O6b atoms.

### 2.4 Morphological and compositional analysis

**Fig. 4:**
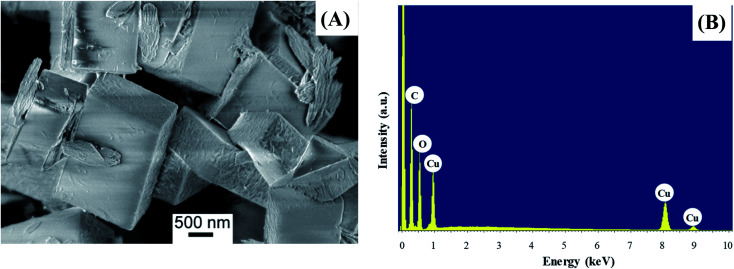
(A) Representative FE-SEM image; (B) corresponding EDXS spectrum.

The morphology of the powder was imaged by means of FE-SEM. The obtained micrograph (Fig. 4A) revealed the presence of two morphologically different types of materials: the first one characterized by large prismatic crystals with nearly micrometric dimensions; the second one constituted by much smaller irregular flakes that tend to aggregate and form larger platelets. In particular, the highly predominant prismatic blocks could be easily attributed to the tetragonal [Cu(1,3-YBDC)]·*x*H_2_O species, while the tiny platelets are attributed to the layered nanosized copper hydroxide and hydroxy-nitrate evidenced in the XRD trace (see above). The compositional purity of the material under the electron beam was investigated by EDXS; the obtained spectrum (Fig. 4B) showed the presence of CKα and OKα peaks, located respectively at energy values of 0.28 and 0.52 keV, as well as of CuLα (0.93 keV), CuKα (8.04 eV), and CuKβ (8.92 eV). No signals from other elements could be detected, the paucity of the N atoms making their signal hidden below the C and O peaks. The obtained Cu/C ratio calculation yielded a value of 0.07, in good agreement with the one obtained by XPS analysis (see below).

**Fig. 5:**
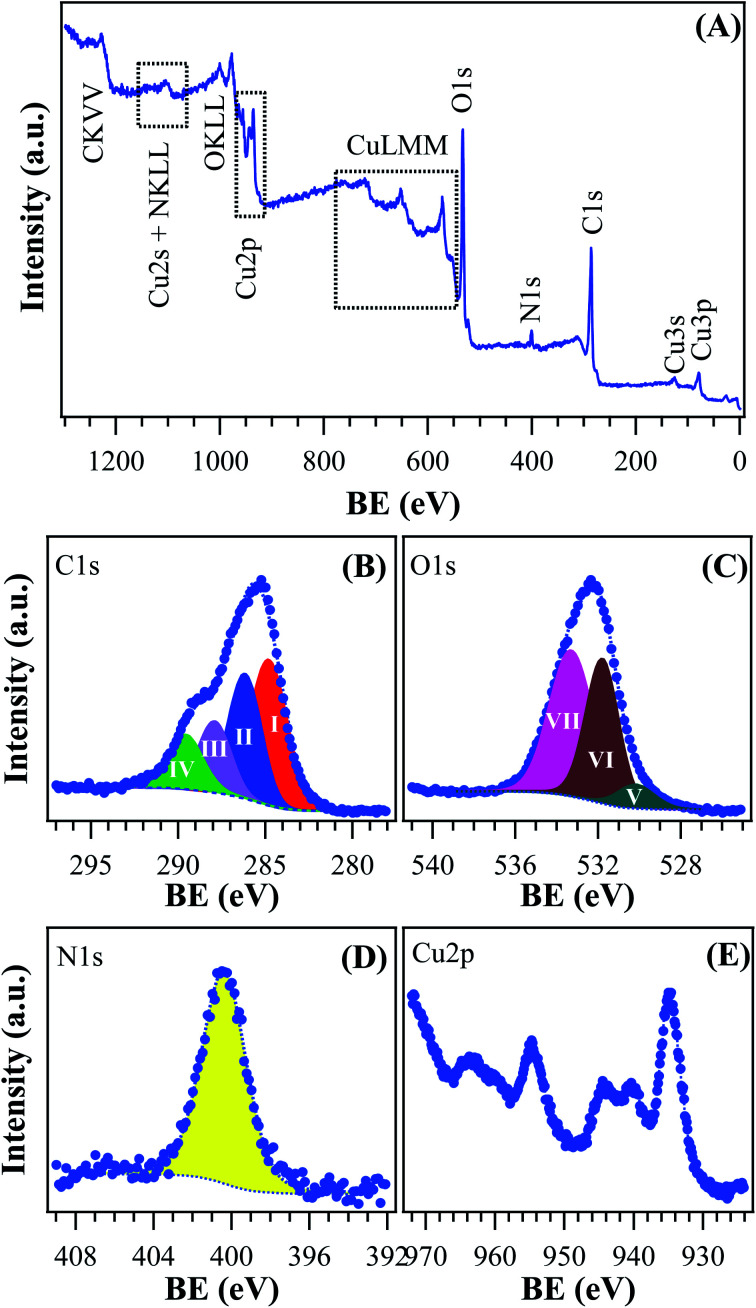
XPS analysis for the target [Cu(1,3-YBDC)]·*x*H_2_O specimen: (A) wide-scan spectrum; (B) C 1s, (C) O 1s, (D) N 1s, (E) Cu 2p photoelectron peaks.

Additional important information was gained by XPS analysis, which was used to characterize material surface chemical composition and element chemical states. The analysis revealed the presence of C, N, O, and Cu, in line with the effective MOF composition (Fig. 5A). Quantitative analyses yielded the following data: C, 58.7 at%; N, 5.2 at%; O, 31.3 at%; Cu, 4.8 at% (Cu/C atomic percentage ratio = 0.08). The C 1s signal could be deconvoluted by means of four contributing components (see Fig. 5B) related to the structure of the MOF framework: (I) BE = 284.8 eV (typical value ≈40% of the total C signal), assigned both to adventitious carbon contaminations and to carbon atoms with homonuclear (all-carbon) contacts only; (II) BE = 286.2 eV (≈30% of the total C area), related mainly to contributions from carbon atoms bound to N and O atoms in the ligand skeleton; (III) BE = 287.9 eV (≈20% of the overall carbon content), attributable to Ar–COOH groups, and (IV) BE = 289.6 eV, attributable to –NCOO– moieties in the branching residue.^29,42,43^ Three bands contributed to the O 1s peak (Fig. 5C): (V), BE = 530.2 eV (typical value ≈8% of the total O signal), resulting from the concurrent contribution of O6x atoms (x = a,b) bound to copper;^29,43–45^ (VI), BE = 531.8 eV (≈40% of the total), due to O atoms bound to C and hydroxyl groups^42,46,47^ and (VII), BE = 533.3 eV, related to carboxylic oxygen atoms and/or a concurrent contribution from adsorbed water.^42,43^ The N 1s peaks (Fig. 5D) showed a single contribution centered at BE = 400.3 eV.^48^

The analysis of copper chemical state (Fig. 5E) required particular attention. The energy positions of Cu 2p_3/2_ and Cu 2p_1/2_ spin–orbit components (BE = 934.8 eV and 954.7 eV, respectively) could be ascribed to Cu(ii) centers in the copper-containing metal–organic framework and in Cu(ii) hydroxides impurities.^15,29,42,43,47^ This attribution was in agreement with the presence of intense shake-up satellites centered at BE values ≈9.0 eV higher than the main spin–orbit components, that are considered as a finger-print for the predominant presence of *d*^9^ copper(ii) centers and are not detected in the case of Cu(i) (*d*^10^, closed-shell).^49,50^

### 2.5 Surface area and pores

**Fig. 6:**
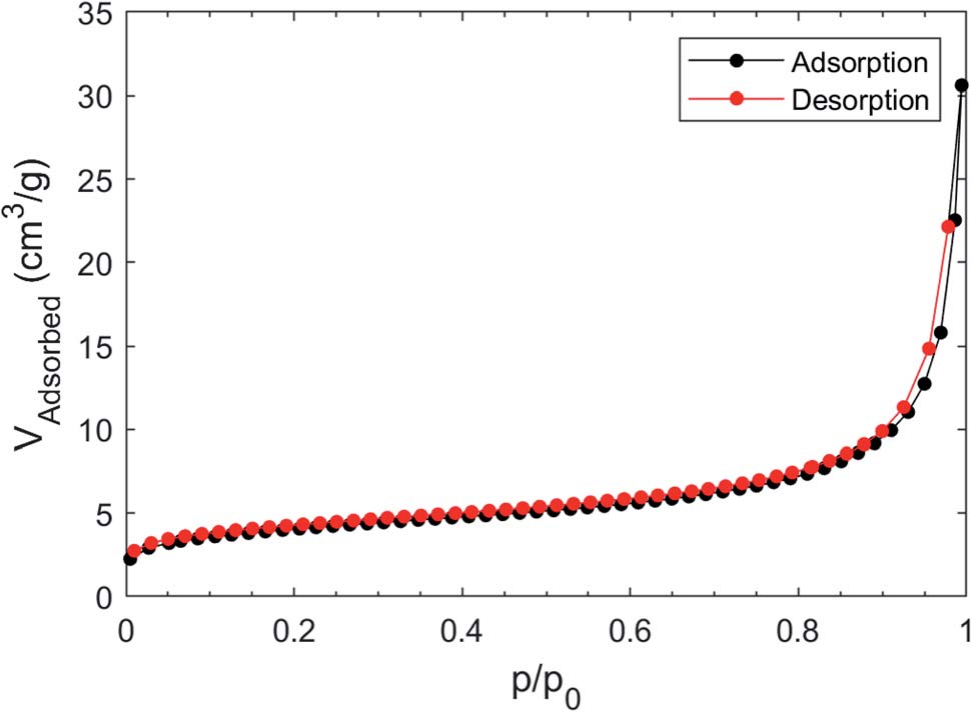
Adsorption and desorption isotherm of N_2_ at 77 K on [Cu(1,3-YBDC)]·*x*H_2_O.

Surface area and pore volume were determined by measuring the adsorption isotherm of nitrogen at 77 K after pre-treating the as made sample under vacuum; the (fully reversible) isotherm, shown in Fig. 6, does not show any gate-opening effect,^51^ confirming the absence of flexibility of the Cu(ii)-isophthalate framework and of its many congeners.^39^

The BET model fits the isotherm slightly better than Langmuir in the low pressure region (Langmuir *R*^2^ = 0.99757, BET *R*^2^ = 0.99986); the Langmuir surface area was found to be 18.4 ± 0.9 m^2^ g^−1^, while the BET surface area was 14.5 ± 0.8 m^2^ g^−1^. The estimated pore volume was 46 mm^3^ g^−1^, while that derived from mercury^52^ was about two times larger (105 mm^3^ g^−1^).

The reported BET for Cu–BTC MOFs are usually above 1000 m^2^ g^−1^,^53^ however the presence of ligands that offer and extra coordination to Cu can significantly drop the surface area to 100 m^2^ g^−1^ or below.^54,55^ Using the computational approach proposed by Düren *et al.*^56^ we have computed, for an idealized ordered and anhydrous crystal phase (such as those provided in the separate *a* and *b* models cited above), a rather small specific surface area of the internal voids (of only 100 m^2^ g^−1^), accounting for 17.1% of the crystal volume. Given that this approach usually provides an estimate of an upper limit (experimental values being often less than one half of what predicted), our measurements are roughly in line with what expected, and possibly further lowered by the occluding effect of coprecipitated nanosized Cu hydroxide and hydroxonitrates lowering the channel accessibility. As a reference values for isostructural compound, we have taken the (pyridine free) copper isophthalate easily derived from the published coordinates of the MOF presented in literature; in this case, SSA = 1509 m^2^ g^−1^ and void percentage = 49.9%.^57^

Using these values and normalizing to the number of Cu_2_ dumbbells in the structure of [Cu(1,3-YBDC)], the size of the MOF cavities, arranged in poly-hourglass shaped channels running along a and b, is only 141 Å^3^. However, taking into account the approximate volume of the tetrachloroaurate anion [the Au(0) precursor, ≈142 Å^3^] and the tabulated size of a single Au atom [≈43(2) Å^3^],^58^ we hardly envisage that the MOF could be used for capturing gold species inside the material, particularly, due to charge-balancing effects by additional counterions and by the expected presence of a solvation sphere. Thus, Au species may bind primarily on the MOF external surface, which is covered by a dense and even array of protruding propargyl-carbamate residues. Considering the size of crystalline domain estimated by the XRD model (of the order of 100 nm for isotropic particles), nearly 2% of the ligands lie on the crystal surface, possibly acting as catalytic sites.

## 3. Conclusions

In summary, we showed that the reaction of the novel 5-substituted organic linker 5-(2-{[(prop-2-yn-1-yloxy)carbonyl]-amino}ethoxy)isophthalic acid, bearing a propargyl carbamate substituent, with copper nitrate in refluxing 2-propanol leads to a new copper-based MOF [Cu(1,3-YBDC)]·*x*H_2_O in high yields. The novel material was fully characterized by many analytical techniques. Synchrotron X-ray diffraction data, in particular, revealed that [Cu(1,3-YBDC)]·*x*H_2_O contains a complex network of 5-substituted isophthalate anions coordinated to Cu(ii) centers belonging to the common paddlewheel dimeric structure with a short Cu⋯Cu distance of 2.633(4) Å. Quite unexpectedly, the apical atom in the paddlewheel structure belongs to the carbonyl atom of the propargyl carbamate functionality, which is present with two equally populated alternative chain conformations. Such extra coordination by the propargyl carbamate groups drastically reduces the MOF porosity, as also confirmed by BET measurements. Although this evidence suggests that the internal pores of the material are available only to a small extent to host reactive gold species, this material can nonetheless be envisaged to be used for generation and anchoring of reduced gold species on the MOF surface, due to its dense, even array of propargyl-carbamate residues branching out therefrom and acting as binding sites able to capture Au(iii) ions and subsequently forming Au(0) clusters.

## 4. Experimental Section

### 4.1 Materials

Cu(NO_3_)_2_ · 2.5H_2_O (98%) and all chemicals employed in the synthesis were of analytical reagent grade and purchased from Sigma-Aldrich. Details regarding the synthesis and characterization of the new dicarboxylic linker 1,3-H_2_YBDC described in Scheme 1 are reported in the ESI.†

### 4.2 *Ab initio* crystal structure solution from synchrotron X-ray diffraction data

Synchrotron data for structure solution of the [Cu(1,3-YBDC)]·*x*H_2_O phase (*x* ∼ 2) were collected in transmission mode on polycrystalline powders loaded into a G50 glass capillary (0.5 mm in diameter) at the high-angle resolution Material Science MS-X04SA beamline of the Swiss Light Source (SLS) of the Paul Scherrer Institute. The operational wavelength, calibrated with the use of a silicon standard (NIST SRM 640c), was 0.56377 Å. The beamline description and the available set of optics and the Si microstrip 1D Mythen detector, with nominal step-size of 0.0036°, are detailed in ref. 59. Cell determination, structure solution and refinement procedures are described in the ESI.† Further analysis of the experimental diffraction pattern suggested that contaminant nanocrystalline phases were also present, and attributed to Cu_2_(OH)_3_(NO_3_) and Cu(OH)_2_. Fractional atomic coordinates and crystal structure details of [Cu(1,3-YBDC)]·*x*H_2_O were deposited with the CCDC (CSD code 2054304).

#### Crystal data for [Cu(1,3-YBDC)]·2H_2_O

C_14_H_15_CuNO_9_, fw = 404.82 g mol^−1^, tetragonal, *P*4/*ncc*, *a* = *b* = 18.5281(2), *c* = 19.2204(4) Å, *V* = 6598.2(2) Å^3^, *Z* = 16, *ρ*_calc_ = 1.629 g cm^−3^, *μ* = 7.1 cm^−1^; *R*_p_ and *R*_wp_, 0.070 and 0.085 respectively, for 7708 data collected in the 2.25–30.00° 2*θ* range. *R*_Bragg_ = 0.045.

### 4.3 Instrumental characterization

Detailed information regarding instruments and methods employed for Electron Spray Ionization Mass Spectrometry (ESI-MS), Nuclear Magnetic Resonance (NMR), Attenuated Total Reflectance Fourier Transformed Infrared spectroscopy (ATR-IR), Thermogravimetric (TGA) analyses, and Atomic Absorption Spectroscopy (AAS) are reported in the ESI file.† Elemental C, H, N analysis were obtained at REDOX s.r.l. (Monza, Italy).

Field emission-scanning electron microscopy (FE-SEM) analysis was carried out by means of a Zeiss SUPRA 40VP instrument equipped with an INCAx-act PentaFET Precision spectrometer (Oxford Instruments) for energy dispersive X-ray spectroscopy (EDXS) characterization. The used primary beam acceleration voltages were comprised between 1 kV (for imaging) and 20 kV (for EDXS analyses).

X-ray photoelectron (XPS) characterization was performed using a Perkin-Elmer Φ 5600-ci instrument, at an operating pressure <10^−8^ mbar, using a standard AlKα excitation source (*hν* = 1486.6 eV) and an analysis area with a diameter of 800 μm. Survey scans were acquired in the 0–1300 eV range (187.8 eV pass energy, 0.8 eV per step, 0.02 s per step). Higher resolution scans for the single photopeaks were recorded using the following settings: 58.7 eV pass energy, 0.1 eV per step, 0.05 s per step. Binding energy values (BEs; uncertainty = ±0.2 eV) were corrected for charging by assigning to the adventitious C 1s peak associated with adventitious hydrocarbons a value of 284.8 eV.^60^ After a Shirley-type background subtraction,^61^ curve fitting was carried out by the XPS peak software.^62^ Atomic percentages (at%) were evaluated from integrated peak areas using sensitivity factors supplied by Perkin-Elmer.^42^

Raman spectra were recorded using a HORIBA Jobin Yvon T64000 spectrometer equipped with three monochromators in double subtractive configuration. The spectrometer was coupled to an Olympus BX40 confocal microscope equipped with 100×, 50×, 20×, and 10× objectives, for a lateral resolution lower than 1 μm with the 100× objective. An Ar+ laser emitting at 514.5 nm was used in which its output power was limited in order to avoid sample damaging (70–100 mW) and with long times and accumulations (about 50–60 min for spectrum).

The adsorption isotherm was measured by using a static volumetric apparatus (ASAP 2020, Micromeritics, USA). The absolute total surface area (SA) of the solid powder (>1.5 m^2^) was above the lowest measurable value (0.5 m^2^). The errors on SA were calculated by standard deviation on 5 consecutive measurements. The sample was degassed at 1 × 10^−3^ mbar at 323 K for 2 h prior the measurement and after this operation *ca.* 5% mass loss was observed, in according with TGA. The BET model is usually considered the reference for MOF,^1^ however, in order to obtain the highest agreement between the measured area and the effective geometrical area, the correct interval of pressure must be taken in account;^63^ in our case the optimal pressure range was between 0 and *p*/*p*_0_ < 0.075.

### 4.4 Preparation of [Cu(1,3-YBDC)]·*x*H_2_O

In a 100 mL round-bottom flask, a suspension of 1,3-H_2_YBDC (0.120 g, 0.390 mmol) and Cu(NO_3_)_2_ · 2.5H_2_O (0.163 g, 0.701 mmol) in 2-propanol (15 mL) was refluxed at 83 °C under stirring (300 rpm) for 24 h and then cooled to room temperature. The precipitate was collected by Buchner filtration and washed with 2-propanol (2 × 10 mL). The turquoise microcrystalline powder was then dried in an oven at 70 °C for 24 h, successively kept under vacuum (0.02 bar) for 24 h and stored under nitrogen to give 0.142 g of [Cu(1,3-YBDC)]·*x*H_2_O (yield 90% based on the organic acid and considering the Cu–MOF dihydrate). The reaction can be easily scaled up to produce gram quantities. The elemental analysis of the synthesized sample (C, 42.48; H, 3.59; N, 3.60, Cu, 15.91) is in good agreement with the calculated composition of [Cu(1,3-YBDC)]·2H_2_O (C_14_H_15_NO_9_Cu: C, 41.54; H, 3.73; N, 3.46; Cu, 15.70%).

## Conflicts of interest

There are no conflicts to declare.

## Supplementary Material

RA-011-D1RA02686K-s001

RA-011-D1RA02686K-s002

RA-011-D1RA02686K-s003

RA-011-D1RA02686K-s004

## References

[cit1] Howarth A. J., Peters A. W., Vermeulen N. A., Wang T. C., Hupp J. T., Farha O. K. (2017). Chem. Mater..

[cit2] Stock N., Biswas S. (2012). Chem. Rev..

[cit3] Chughtai A. H., Ahmad N., Younus H. A., Laypkov A., Verpoort F. (2015). Chem. Soc. Rev..

[cit4] Janiak C., Vieth J. K. (2010). New J. Chem..

[cit5] Wang Q., Astruc D. (2020). Chem. Rev..

[cit6] Mohideen M. I. H., Xiao B., Wheatley P. S., McKinlay A. C., Li Y., Slawin A. M. Z., Aldous D. W., Cessford N. F., Düren T., Zhao X., Gill R., Thomas K. M., Griffin J. M., Ashbrook S. E., Morris R. E. (2011). Nat. Chem..

[cit7] Edwards P. , Global Cement Magazine, 2020 (December), pp. 20–24, www.global.cement.com

[cit8] Metal–Organic Framework: From Design to Applications, ed. X.-H. Bu, M. J. Zaworotko and Z. Zhang, Springer, 2020

[cit9] Allendorf M. D., Stavila V. (2015). CrystEngComm.

[cit10] Pettinari C., Marchetti F., Mosca N., Tosi G., Drozdov A. (2017). Polym. Int..

[cit11] Dey C., Kundu T., Biswal B. P., Mallick A., Banerjee R. (2014). Acta Crystallogr., Sect. B: Struct. Sci., Cryst. Eng. Mater..

[cit12] Chui S. S. Y., Lo S. M. F., Charmant J. P. H., Orpen A. G., Williams I. D. (1999). Science.

[cit13] Dawson D. M., Sansome C. E. F., McHugh L. N., McPherson M. J., McCormick McPherson L. J., Morris R. E., Ashbrook S. E. (2019). Solid State Nucl. Magn. Reson..

[cit14] Schlichte K., Kratzke T., Kaskel S. (2004). Microporous Mesoporous Mater..

[cit15] Senthil Kumar R., Senthil Kumar S., Anbu Kulandainathan M. (2013). Microporous Mesoporous Mater..

[cit16] Pöppl A., Kunz S., Himsl D., Hartmann M. (2008). J. Phys. Chem. C.

[cit17] Eddaoudi M., Kim J., Vodak D., Sudik A., Wachter J., O'Keeffe M., Yaghi O. M. (2002). Proc. Natl. Acad. Sci. U. S. A..

[cit18] Li H., Eddaoudi M., O'Keeffe M., Yaghi O. M. (1999). Nature.

[cit19] Lee H. K., Min D., Cho B. Y., Lee S. W. (2004). Bull. Korean Chem. Soc..

[cit20] LaDuca R. L. (2009). Coord. Chem. Rev..

[cit21] Ballarin B., Barreca D., Boanini E., Cassani M. C., Dambruoso P., Massi A., Mignani A., Nanni D., Parise C., Zaghi A. (2017). ACS Sustainable Chem. Eng..

[cit22] Parise C., Ballarin B., Barreca D., Cassani M. C., Dambruoso P., Nanni D., Ragazzini I., Boanini E. (2019). Appl. Surf. Sci..

[cit23] Ballarin B., Cassani M. C., Nanni D., Parise C., Barreca D., Carraro G., Riminucci A., Bergenti I., Morandi V., Migliori A., Boanini E. (2019). Ceram. Int..

[cit24] Ballarin B., Barreca D., Boanini E., Bonansegna E., Cassani M. C., Carraro G., Fazzini S., Mignani A., Nanni D., Pinelli D. (2016). RSC Adv..

[cit25] Gualtieri A. F., Gatta G. Di., Arletti R., Artioli G., Ballirano P., Cruciani G., Guagliardi A., Malferrari D., Masciocchi N., Scardi P. (2019). Period. Mineral..

[cit26] Wenk H. R., Van Houtte P. (2004). Rep. Prog. Phys..

[cit27] Kulesza J., Silva Barros B., Alves Júnior S., Fernandes De Oliveira C. A., De Araújo Melo D. M., Chojnacki J. (2014). Mater. Chem. Phys..

[cit28] Nakamoto K. , Infrared and Raman Spectra of Inorganic and Coordination Compounds, John Wiley & Sons, Hoboken, New Jersey, 6th edn, 2009

[cit29] Azhar M. R., Hussain G., Tade M. O., Silvester D. S., Wang S. (2020). ACS Appl. Nano Mater..

[cit30] Feng L., Chen Z., Zeller M., Luck R. L. (2013). Inorg. Chim. Acta.

[cit31] Zhang Y. M., Yang L. Z., Lin Q., Wei T. B. (2005). Transition Met. Chem..

[cit32] Cortés-Súarez J., Celis-Arias V., Beltrán H. I., Tejeda-Cruz A., Ibarra I. A., Romero-Ibarra J. E., Sánchez-González E., Loera-Serna S. (2019). ACS Omega.

[cit33] Choi J. S., Bae J., Lee E. J., Jeong N. C. (2018). Inorg. Chem..

[cit34] Prestipino C., Regli L., Vitillo J. G., Bonino F., Damin A., Lamberti C., Zecchina A., Solari P. L., Kongshaug K. O., Bordiga S. (2006). Chem. Mater..

[cit35] Dong Z., Mi Z., Shi W., Jiang H., Zheng Y., Yang K. (2017). RSC Adv..

[cit36] Fukuda M., Koga N. (2018). J. Phys. Chem. C.

[cit37] Schildermans I., Mullens J., Van der Veken B. J., Yperman J., Franco D., Van Poucke L. C. (1993). Thermochim. Acta.

[cit38] Jurczak E., Mazurek A. H., Szeleszczuk Ł., Pisklak D. M., Zielińska-Pisklak M. (2020). Pharmaceutics.

[cit39] Köberl M., Cokoja M., Herrmann W. A., Kühn F. E. (2011). Dalton Trans..

[cit40] Bertolotti F., Forni A., Gervasio G., Marabello D., Diana E. (2012). Polyhedron.

[cit41] Abourahma H., Bodwell G. J., Lu J., Moulton B., Pottie I. R., Walsh R. B., Zaworotko M. J. (2003). Cryst. Growth Des..

[cit42] Moulder J. F. , Stickle W. F., Sobol P. E. and Bomben K. D., Handbook of X-ray Photoelectron Spectroscopy, Perkin Elmer Corporation, Eden Prairie, MN, USA, 1992

[cit43] http://srdata.nist.gov/xps

[cit44] Barreca D., Gasparotto A., Tondello E. (2007). Surf. Sci. Spectra.

[cit45] Barreca D., Gasparotto A., Maccato C., Tondello E., Lebedev O. I., Van Tendeloo G. (2009). Cryst. Growth Des..

[cit46] Li X., Wan J., Ma Y., Zhao J. R., Wang Y. (2020). Appl. Surf. Sci..

[cit47] Zhao X., Wu W., Jing G., Zhou Z. (2020). Environ. Pollut..

[cit48] Taher A., Kim D. W., Lee I. M. (2017). RSC Adv..

[cit49] Barreca D., Carraro G., Gasparotto A. (2009). Surf. Sci. Spectra.

[cit50] Chen C., Wu T., Yang D., Zhang P., Liu H., Yang Y., Yang G., Han B. (2018). Chem. Commun..

[cit51] Sakata Y., Furukawa S., Kondo M., Hirai K., Horike N., Takashima Y., Uehara H., Louvain N., Meilikhov M., Tsuruoka T., Isoda S., Kosaka W., Sakata O., Kitagawa S. (2013). Science.

[cit52] MacRae C. F., Sovago I., Cottrell S. J., Galek P. T. A., McCabe P., Pidcock E., Platings M., Shields G. P., Stevens J. S., Towler M., Wood P. A. (2020). J. Appl. Crystallogr..

[cit53] Lee Y. R., Kim J., Ahn W. S. (2013). Korean J. Chem. Eng..

[cit54] Ghamari Kargar P., Aryanejad S., Bagherzade G. (2020). Appl. Organomet. Chem..

[cit55] Wang S., Wang L., Zhu Y., Song Y. (2020). Spectrochim. Acta, Part A.

[cit56] Düren T., Millange F., Férey G., Walton K. S., Snurr R. Q. (2007). J. Phys. Chem. C.

[cit57] Bourne S. A., Lu J., Mondal A., Moulton B., Zaworotko M. J. (2001). Angew. Chem., Int. Ed..

[cit58] Hofmann D. W. M. (2002). Acta Crystallogr., Sect. B: Struct. Sci..

[cit59] Willmott P. R., Meister D., Leake S. J., Lange M., Bergamaschi A., Böge M., Calvi M., Cancellieri C., Casati N., Cervellino A., Chen Q., David C., Flechsig U., Gozzo F., Henrich B., Jäggi-Spielmann S., Jakob B., Kalichava I., Karvinen P., Krempasky J., Lüdeke A., Lüscher R., Maag S., Quitmann C., Reinle-Schmitt M. L., Schmidt T., Schmitt B., Streun A., Vartiainen I., Vitins M., Wang X., Wullschleger R. (2013). J. Synchrotron Radiat..

[cit60] Briggs D. and Seah M. P., Practical surface analysis: Auger and X-ray photoelectron spectroscopy, John Wiley & Sons, New York, 2nd edn, 1990

[cit61] Shirley D. A. (1972). Phys. Rev. B: Condens. Matter Mater. Phys..

[cit62] https://xpspeak.software.informer.com/4.1/

[cit63] Gómez-Gualdrón D. A., Moghadam P. Z., Hupp J. T., Farha O. K., Snurr R. Q. (2016). J. Am. Chem. Soc..

