# Supplementary material for: A Cu(ii)-MOF based on a propargyl carbamate-functionalized isophthalate ligand
Source: RSC Adv. 2021 Jun 7;11(33):20429–38. doi: 10.1039/d1ra02686k (PMC9034020; doi:10.1039/d1ra02686k)
Supplement: RA-011-D1RA02686K-s004 [file RA-011-D1RA02686K-s004.pdf]

Electronic Supplementary Material (ESI) for RSC Advances.
This journal is © The Royal Society of Chemistry 2021

Supporting Information for:

A Cu(II)-MOF based on a propargyl carbamate-functionalized isophtalate ligand

Maria Cristina Cassani,[a] Francesca Gambassi,[a] Barbara Ballarin,[a] Daniele Nanni,[a*] Ilaria Ragazzini,[a] Davide Barreca,[b] Chiara Maccato,[c] Antonietta Guagliardi,[d] Norberto Masciocchi,[e] Alessandro Kovtun,[f] Katia Rubini,[g] and Elisa Boanini[g]*

[a]Dept. of Industrial Chemistry "Toso Montanari", Bologna University, Viale Risorgimento 4, 40136, Bologna, Italy; [b]CNR-ICMATE and INSTM, Dept. of Chemical Sciences, Padova University, Via Marzolo 1, 35131, Padova, Italy; [c]Dept. of Chemical Sciences, Padova University and INSTM, Via Marzolo 1, 35131, Padova, Italy; [d]Institute of Crystallography and To.Sca.Lab, National Research Council, via Valleggio 11, 22100 Como, Italy; [e]Dept. of Science and High Technology & To.Sca.Lab., University of Insubria, via Valleggio 11, 22100 Como. Italy; [f]Institute of Organic Synthesis and Photoreactivity, (CNR-ISOF), Via P. Gobetti 101, 40129 Bologna, Italy; [g]Dept. of Chemistry "Giacomo Ciamician", Bologna University, Via Selmi 2, 40126, Bologna, Italy.

S.1 Synthesis and Characterization of 5-(2-{[(prop-2-yn-1-yloxy)carbonyl]-amino}ethoxy)isophthalic acid — ***p. S2***
- *S.1.1 General Information: materials and instruments*
- *S.1.2 Synthetic Procedures*

S.2 Characterization of [Cu(1,3-YBDC)]·xH$_2$O — ***p. S11***
- *S.2.1 Spectroscopic investigation*
- *S.2.2 X-ray diffraction investigation*
- *S.2.3 Atomic absorption spectroscopy (AAS)*
- *S.2.4 Ab-initio crystal structure solution from synchrotron X-ray diffraction data*

Supplementary References — ***p. S16***

*To whom correspondence should be addressed. E-mail: maria.cassani@unibo.it (M.C.C.); tel: +39 051 2093700; daniele.nanni@unibo.it (D.N.); tel: +39 051 2093623.

§ S.1 Synthesis and Characterization of 5-(2-{[(prop-2-yn-1-yloxy)carbonyl]-amino}ethoxy)isophthalic acid

S.1.1. General Information: materials and instruments

Materials. All reagents and solvents were used as received, ultrapure water purified with the Milli-Q plus system (Millipore Co, resistivity over 18 MΩ cm) was used in all cases. The reactions were monitored by thin-layer chromatography (TLC) on highly purified Silica on TLC-PET foils (with fluorescent indicator 254 nm, Fluka). ESI-MS analyses were performed by direct injection of methanol solutions using a WATERS ZQ 4000 mass spectrometer; working temperature: 80 ÷ 100 °C; working concentrations: ca. 10^{-8} g/L; Cone Voltage: 10 ÷ 30 V; working flow: 10 μL/min. The NMR spectra were recorded at 298 K using a Varian MercuryPlus VX 400 (^{1}H, 399.9; ^{13}C, 100.6 MHz); spectra were referenced internally to residual solvent resonances and were recorded at 298 K for characterization purposes; full ^{1}H and ^{13}C NMR assignments were done using standard Varian pulse sequences. Spectra have been edited with the software MestReNova Version: 14.1.0-24037, 2019 Mestrelab Research S.L. Abbreviations: s = singlet, d = doublet, t = triplet, m = multiplet, bs = broad singlet. ATR-FTIR analyses were performed with a Perkin Elmer Spectrum Two spectrophotometer, equipped with a Universal ATR accessory, in the range 4000-600 cm^{-1} with a resolution of 0.5 cm^{-1}. Abbreviations: ν = stretching, δ = bending. Thermogravimetric analyses were carried out using a Perkin Elmer TGA-7. The samples (initial weight ≈10 mg) were heated in a platinum crucible at a rate of 10 °C min^{-1} from room temperature to 900 °C.

S.1.2 Synthetic Procedures

Synthesis of prop-2-yn-1-yl *N*-(2-bromoethyl)carbamate (3)[1]

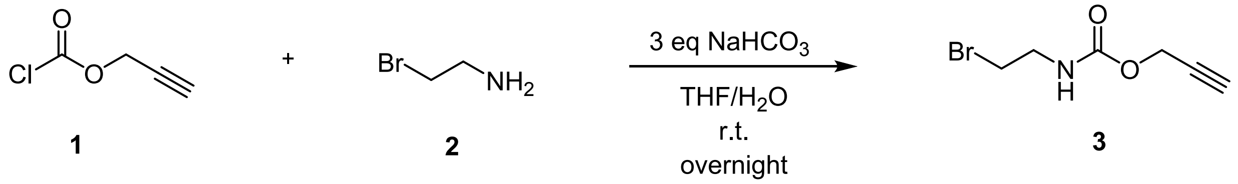

To a solution of 2-bromoethylamine hydrobromide (**2**, 17.8 g, 86.9 mmol) in THF/H_2O (214 mL/171 mL), cooled to 0 °C, $NaHCO_3$ (21.9 g, 261 mmol, 3 eq) was added, followed by propargyl chloroformate (**1**, 10.0 mL, 102.5 mmol, 1.18 eq) added dropwise. The solution was stirred at r.t. overnight. THF was evaporated and the aqueous layer was extracted with ethyl acetate (4 × 20 mL). The organic layer was washed with HCl 1.0 M (2 × 10.0 mL) and brine (2 × 10.0 mL), then dried with $MgSO_4$, filtered and concentrated in vacuum to obtain a colourless oil (**3**, 15.5 g, 75.2 mmol, yield 86.5%). The crude material was used without any further purification. Rf_1 = 0.54, Rf_2 = 0.12, Rf_3 = 0.48 (1:2 petroleum ether/Et_2O).

ESI-MS(+) (MeOH, m/z): 228 (100) $[C_6H_8NO_2Br + Na]^+$.

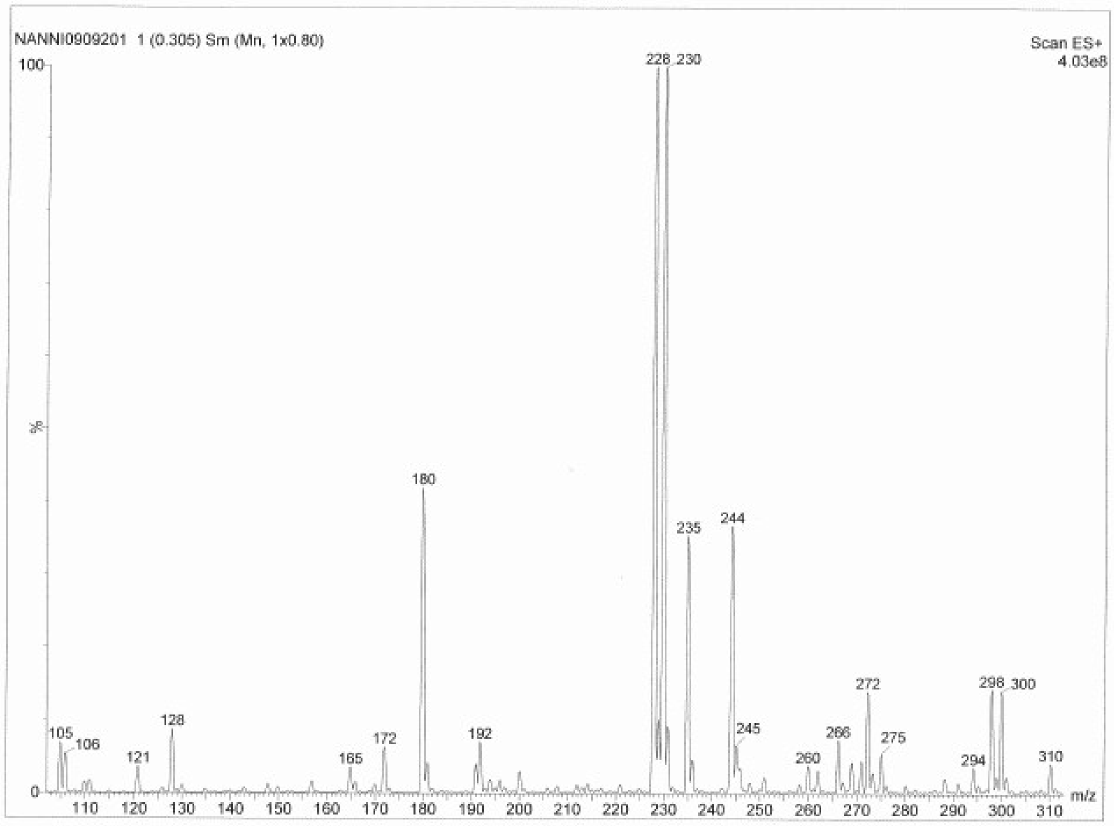

Figure S1. ESI-MS(+) spectrum of **3**.

^{1}H-NMR (400 MHz, $CDCl_3$): δ 5.51 (bs, 1H, NH^{3}), 4.65 (d, $^{4}J_{H,H}$ = 2.4 Hz, 2H, -OCH_2**4**-), 3.55 (m, 2H, -CH_2^{2}N(H)-), 3.44 (t, $^{3}J_{H,H}$ = 6.2 Hz, 2H, $BrCH_2$**1**-), 2.48 (t, $^{4}J_{H,H}$ = 2.4 Hz, 1H, -C≡CH^{5}).

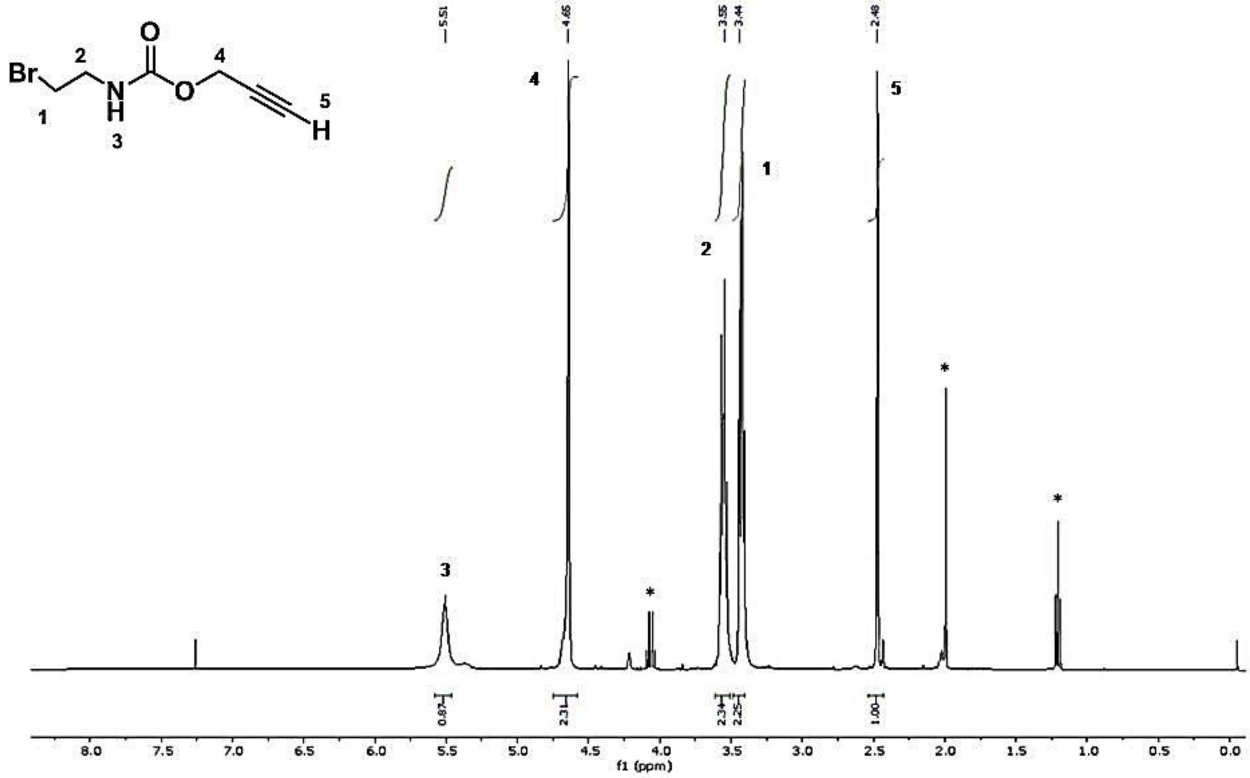

Figure S2. ^{1}H-NMR spectrum of **3.** Signals labelled with an asterisk are relative to the solvent ethyl acetate.

^{13}C-NMR ($CDCl_3$, 100.6 MHz): δ 155.25 (-NHC^{3}(O)O-), 77.97 (-C^{5}≡CH), 74.82 (-C≡C^{6}H), 52.72 (-O$C^{4}H_2$C≡CH), 42.79 (-$C^{2}H_2$NH-), 32.22 (Br$C^{1}H_2$-) ppm.

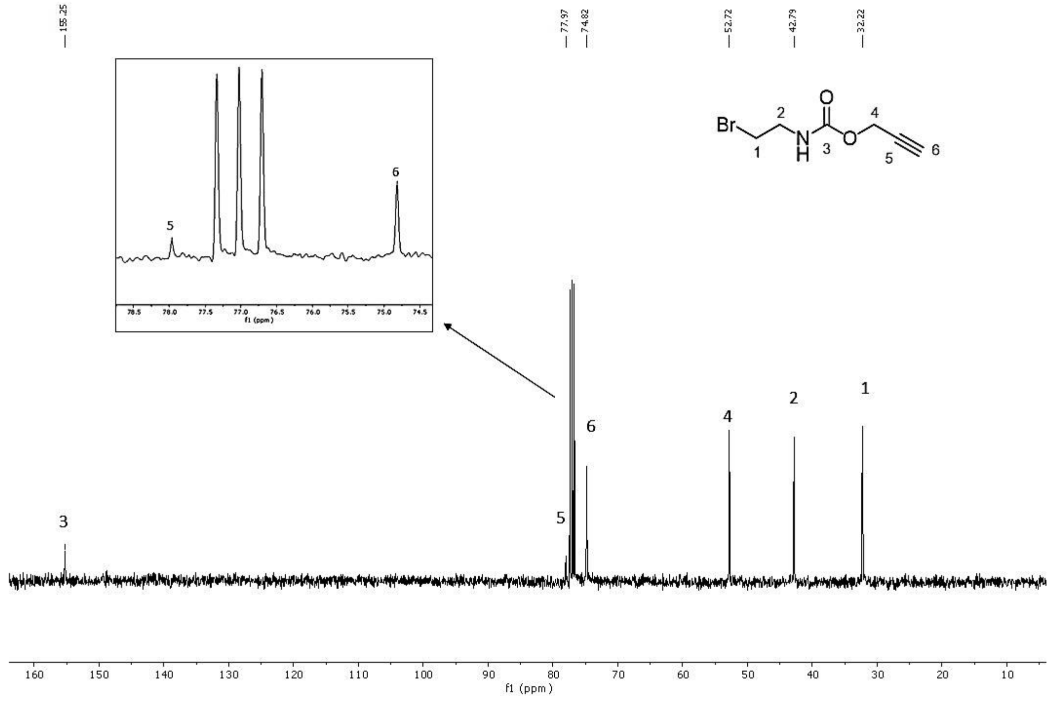

Figure S3. ^{13}C-NMR spectrum of **3**.

ATR-FTIR spectra of neat 3 (cm^{-1}): 3331 (ν NH, m), 3293 (ν ≡C-H, m), 2949 (ν C-H aliphatic, w), 2131 (ν -C≡C-, w), 1703 (ν -C(O)NH- carbamate, s), 1520 (δ NH, s), 1245 (ν C-O, s).

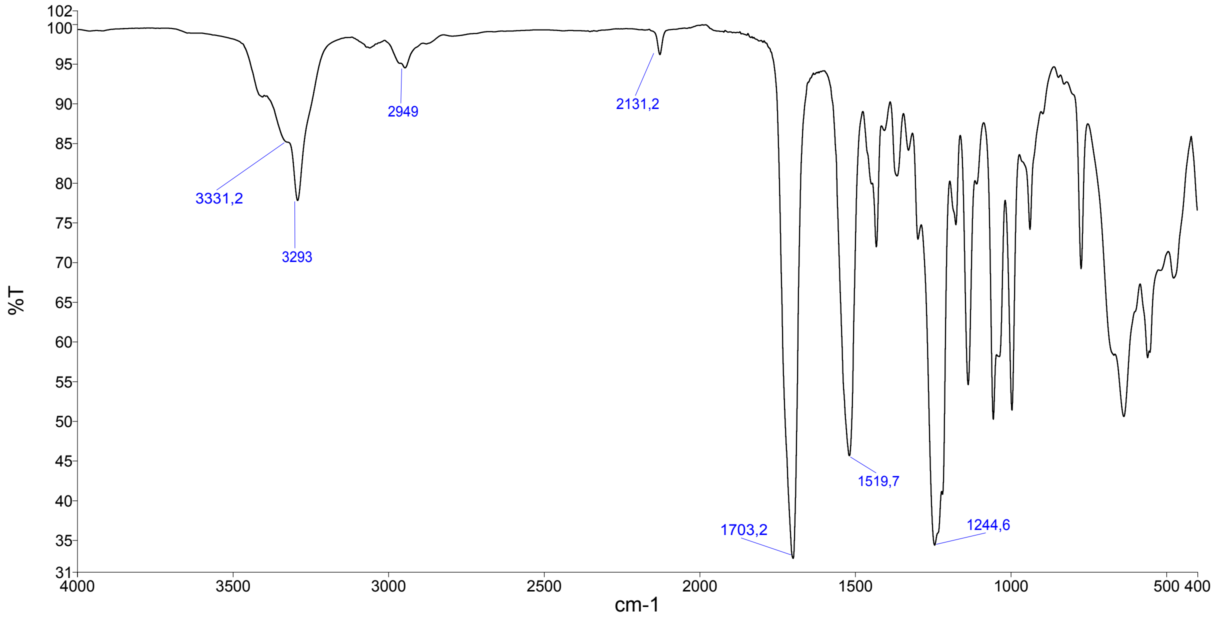

Figure S4. IR-ATR spectrum of **3**.

Synthesis of dimethyl 5-(2-{[(prop-2-yn-1-yloxy)carbonyl]amino}ethoxy)isophthalate (5)[2]

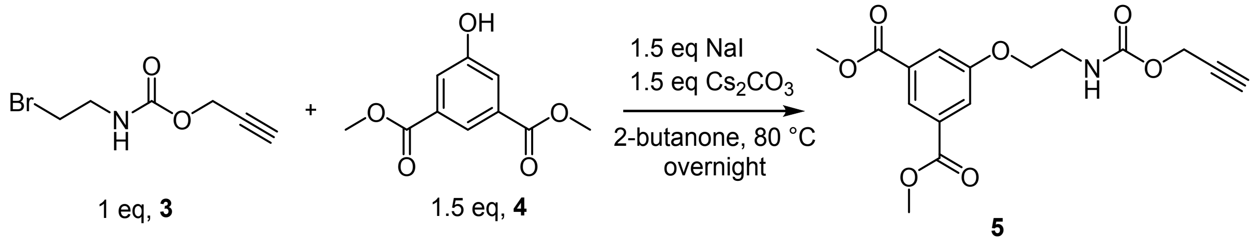

A solution of prop-2-ynyl *N*-(2-bromoethyl)carbamate (**3,** 3.00 g, 14.6 mmol) in butanone (50 mL) was treated with dimethyl 5-hydroxyisophthalate (**4,** 4.60 g, 21.9 mmol), NaI (3.28 g, 21.9 mmol), and Cs_2CO_3 (7.14 g, 21.9 mmol), and the resulting suspension was vigorously stirred at 80 °C overnight. The cooled reaction mixture was first concentrated under vacuum, then diluted with CH_2Cl_2 (50 mL) and subsequently washed with H_2O and then with NaOH 10% v/v. After drying the organic phase with $MgSO_4$, the solvent was removed to afford **5** as a white solid (4.08 g, 12.19 mmol, yield 83.5%). Rf_3 = 0.48, Rf_4 = 0.30, Rf_5 = 0.20 (1:2 petroleum ether/Et_2O). M.p. = 99-102 °C. Soluble at r.t. in $CHCl_3$, CH_2Cl_2, THF, Et_2O, soluble in boiling methanol, ethanol and isopropanol.

ESI-MS(+) (MeOH, m/z): 358 (100) $[C_{16}H_{17}NO_7 + Na]^+$.

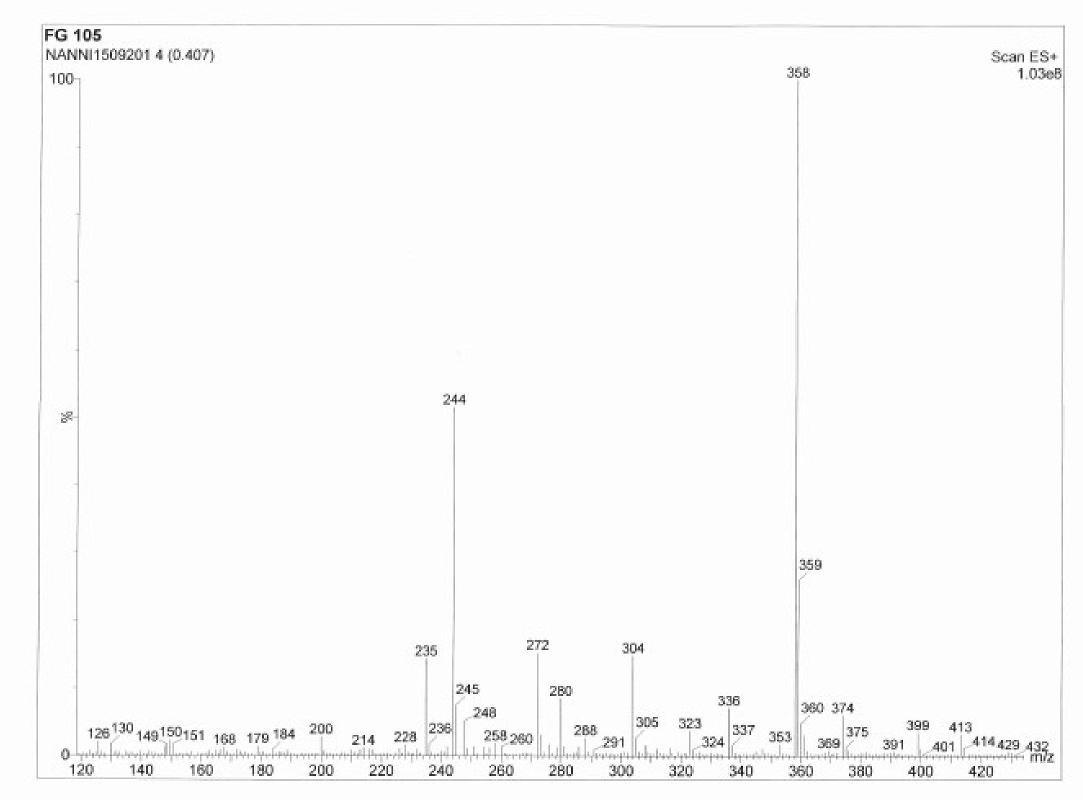

Figure S5: ESI-MS(+) spectrum of **5**.

^{1}H-NMR ($CDCl_3$, 399.9 MHz) δ: 8.30 (t, $^4J_{H,H}$ =1.9 Hz, 1H, Ar-H^2), 7.73 (d, $^4J_{H,H}$ =1.9 Hz, 2H, Ar-H^3), 5.24 (bs, 1H, NH^6), 4.70 (d, $^4J_{H,H}$ = 2.4 Hz, 2H, -$OCH_2{}^7C{\equiv}CH$), 4.13 (t, $^3J_{H,H}$ = 6.8 Hz, 2H, -$OCH_2{}^4$-), 3.94 (s, 6H, $OCH_3{}^1$), 3.63 (m, 2H, -$CH_2{}^5NH$), 2.47 (t, $^4J_{H,H}$ = 2.4 Hz, 1H, -$C{\equiv}CH^8$).

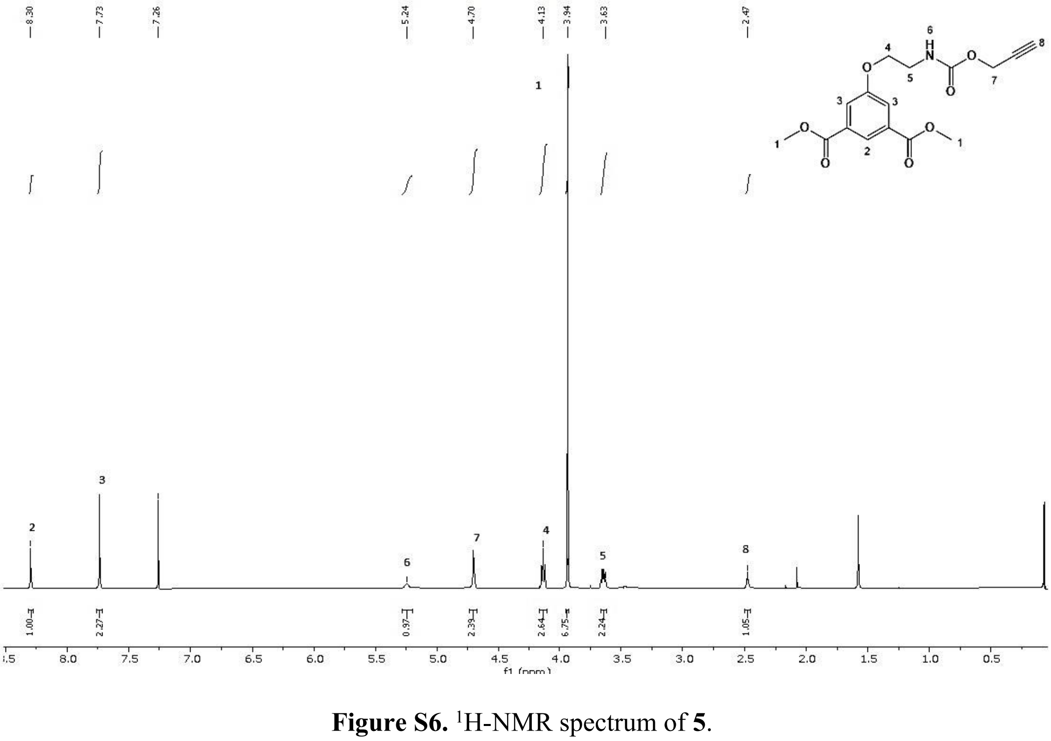

Figure S6. ^{1}H-NMR spectrum of **5**.

^{13}C-NMR ($CDCl_3$, 100.6 MHz) δ: 165.90 (Ar-C^{2}(O)OMe), 158.43 (ArC^{6}O-), 155.44 (-N(H)C^{9}(O)O-), 131.81 (Cq, Ar), 123.36 (CH, Ar), 119.67 (CH, Ar), 78.04 (-C^{11}≡CH), 74.70 (-C≡C^{12}H), 67.35 (-O$C^{7}H_2$-),), 52.58 (-O$C^{10}H_2$-C≡CH), 52.40 (-O$C^{1}H_3$), 40.45 (-$C^{8}H_2$N(H)-) ppm.

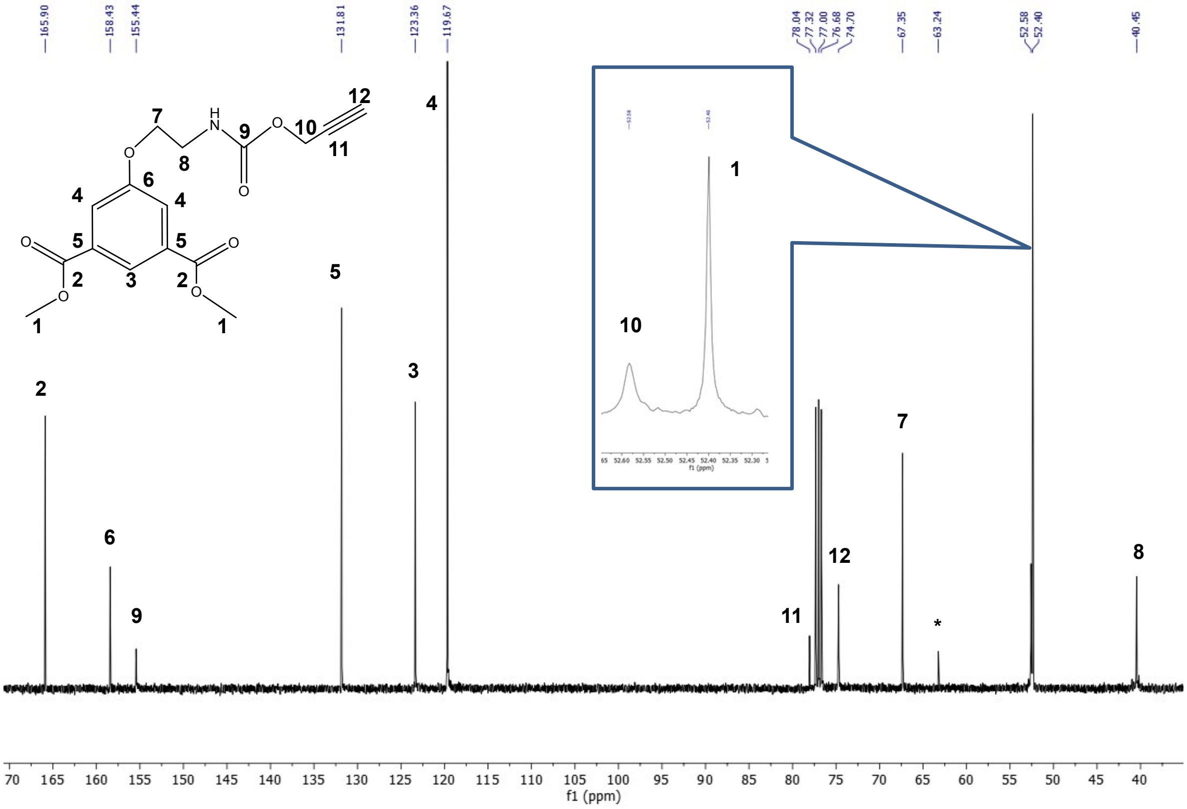

Figure S7. ^{13}C-NMR spectrum of **5.** The signal labelled with an asterisk is relative to a solvent impurity.

ATR-FTIR spectrum of neat 5 (cm^{-1}): 3373 cm^{-1} (ν NH, s), 3258 (ν ≡C-H, s), 3078 – 2840 (ν C-H aliphatic and aromatic, m), 2132 (ν -C≡C-, w), 1738 (ν -C(O)O- carboxylate, s), 1714 (ν -C(O)NH- carbamate, vs), 1544 (δ NH, s), 1243 (ν C-O, vs).

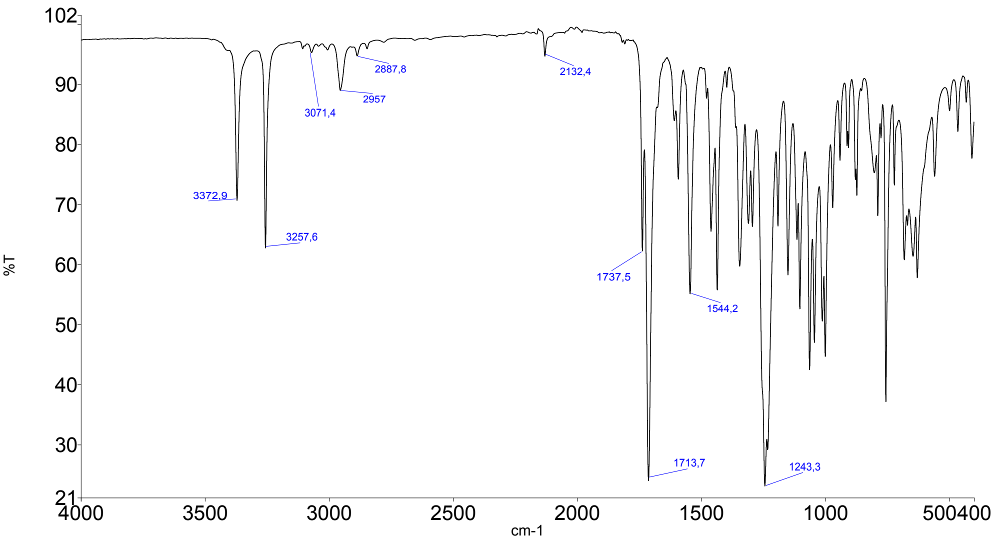

Figure S8. IR-ATR spectrum of **5**.

Synthesis of 5-(2-{[(prop-2-yn-1-yloxy)carbonyl]amino}ethoxy)isophthalic acid (6)[2]

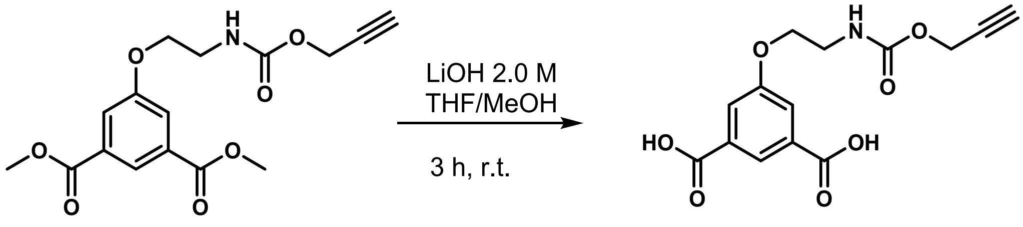

5 **6**

Dimethyl 5-(2-(((prop-2-yn-1-yloxy)carbonyl)amino)ethoxy)isophthalate (**5**, 2.42 g, 7.20 mmol) was dissolved in a 1:1 v/v mixture of THF/methanol (60 mL) and treated with 18 mL of 2.0 M aqueous solution of LiOH. After stirring for 3 h, the solvents were evaporated and the residue was treated with 1.0 M aqueous HCl (20 mL) and extracted with ethylacetate. The organic phase was dried with $MgSO_4$ and evaporated to obtain the pure desired dicarboxylic acid derivative **6** (2.15 g, 7.00 mmol, yield 97%). M.p.= 217-218 °C. Soluble at r.t. in ethylacetate, DMSO, and acetone; soluble in boiling methanol, ethanol and isopropanol. The product was crystallized from ethanol/water at -18 °C before use.

ESI-MS(+) (MeOH, m/z): 330 (100) $[C_{14}H_{13}NO_7 + Na]^+$, 308 (10) $[C_{14}H_{13}NO_7 + H]^+$.

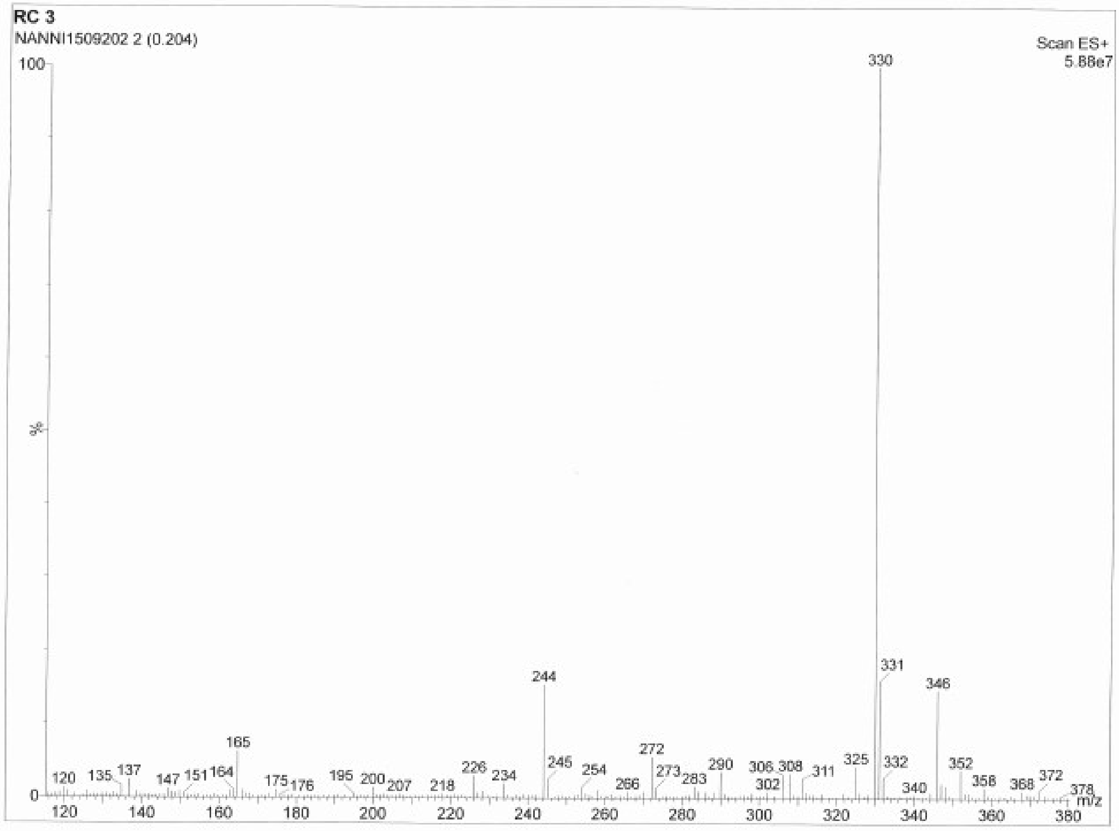

Figure S9. ESI-MS(+) spectrum of **6**.

^{1}H-NMR (acetone-d_6, 399.9 MHz): δ 8.29 (t, $^{4}J_{H,H}$ =1.5 Hz, 1H, Ar-H^{1}), 7.79 (d, $^{4}J_{H,H}$ =1.5 Hz, 2H, Ar-H^{2}), 4.67 (d, $^{4}J_{H,H}$ = 2.4 Hz, 2H, -OCH$_2$^{5}C≡CH), 4.25 (t, $^{3}J_{H,H}$ = 6.8 Hz, 2H, -OCH$_2$^{3}-), 3.60 (t, $^{3}J_{H,H}$ = 6.8 Hz, 2H, -CH$_2$^{4}N(H)-), 2.95 (t, $^{4}J_{H,H}$ = 2.4 Hz, 1H, -C≡CH^{6}) ppm.

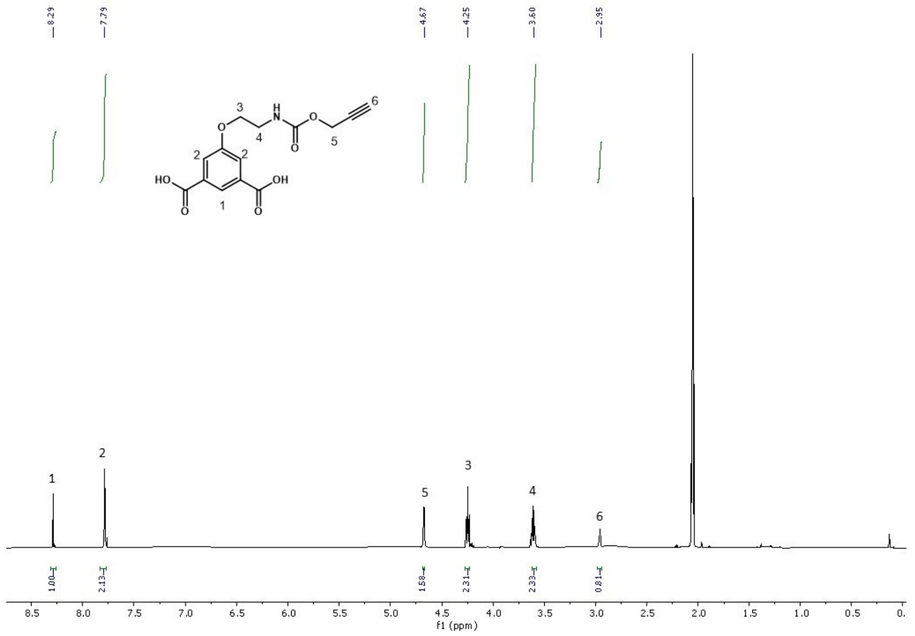

Figure S10. ^{1}H-NMR spectrum of **6**.

^{13}C-NMR (acetone-d_6, 100.6 MHz) δ: 166.73 (Ar*C*^{1}(O)OH), 159.99 (Ar-*C*^{5}O-), 157.00 (-N(H)*C*^{8}(O)O), 133.22 (Cq, Ar), 123.79 (CH, Ar), 120.55 (CH, Ar), 79.73 (-*C*^{10}≡CH), 75.78 (-C≡*C*^{11}H), 68.19 (-O*C*^{6}H$_2$-), 52.48 (-O*C*^{9}H$_2$-C≡CH), 41.08 (-*C*^{7}H$_2$N(H)-) ppm.

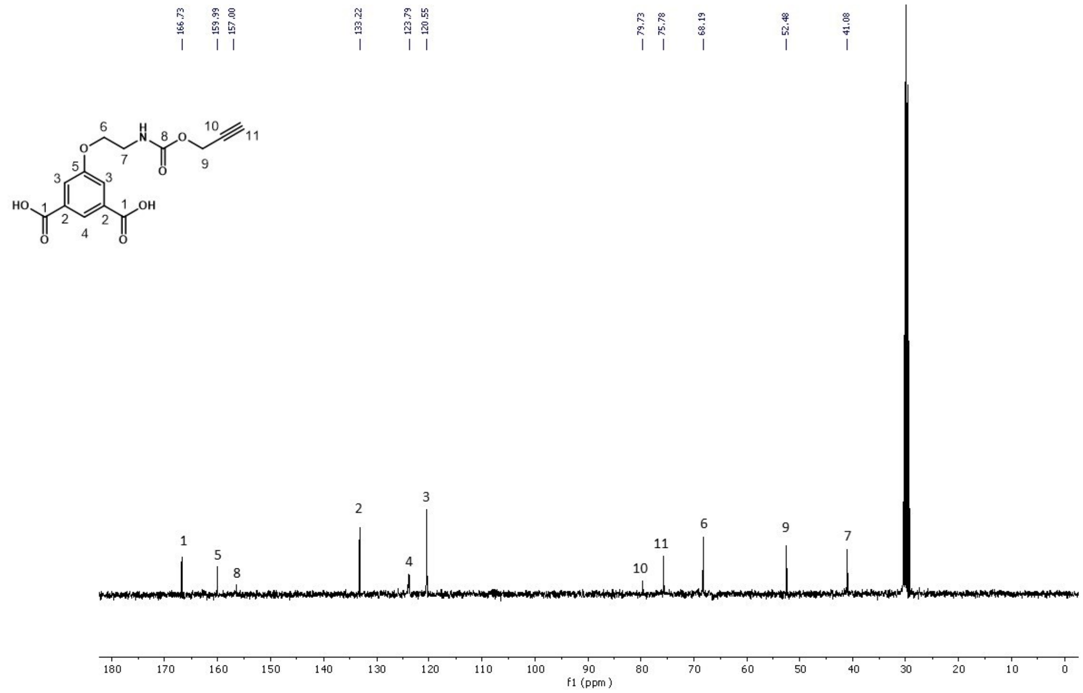

Figure S11. ^{13}C-NMR spectrum of **6**.

ATR-FTIR spectrum of neat 6 (cm^{-1}): 3336 cm^{-1} (ν NH, s), 3304 (ν ≡C-H, s), 3200 – 2800 (ν -OH, broad), 3086-2961 (ν C-H aliphatic and aromatic, m), 2139 (ν -C≡C-, w), 1737 (ν -C(O)O- carboxylate, s), 1687 (ν -C(O)NH- carbamate, vs), 1550 (δ NH, s), 1259 (ν C-O, vs).

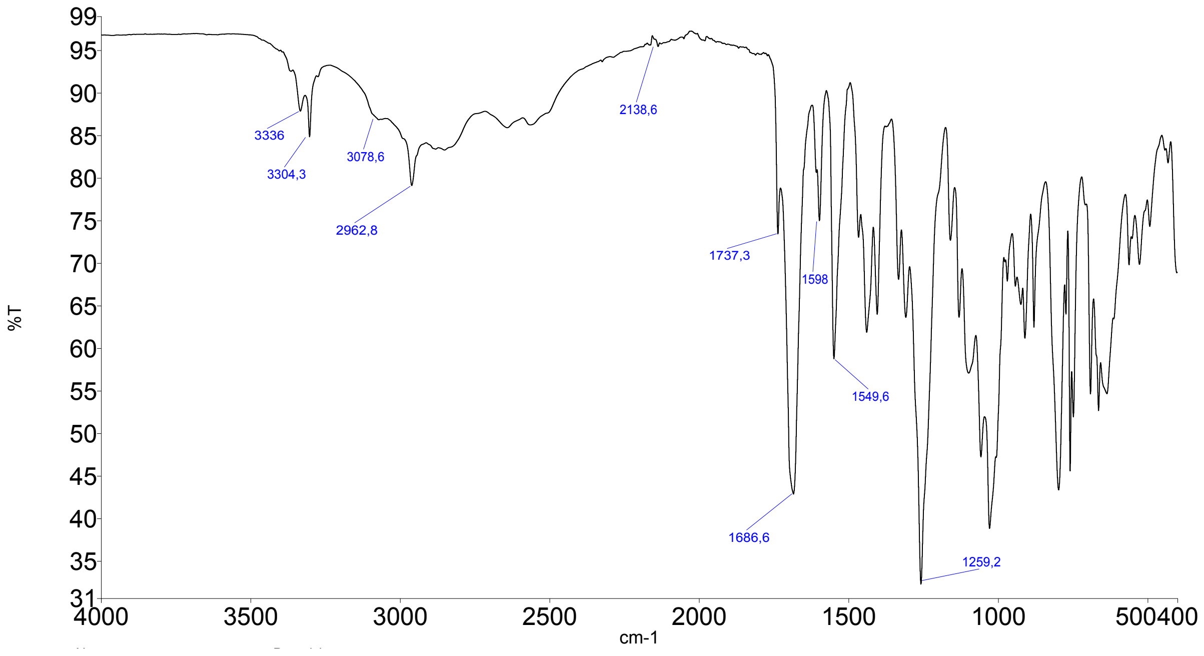

Figure S12. IR-ATR spectrum of **6**.

§ S.2 Characterization of [Cu(1,3-YBDC)]·xH_2O

S.2.1. Spectroscopic investigation

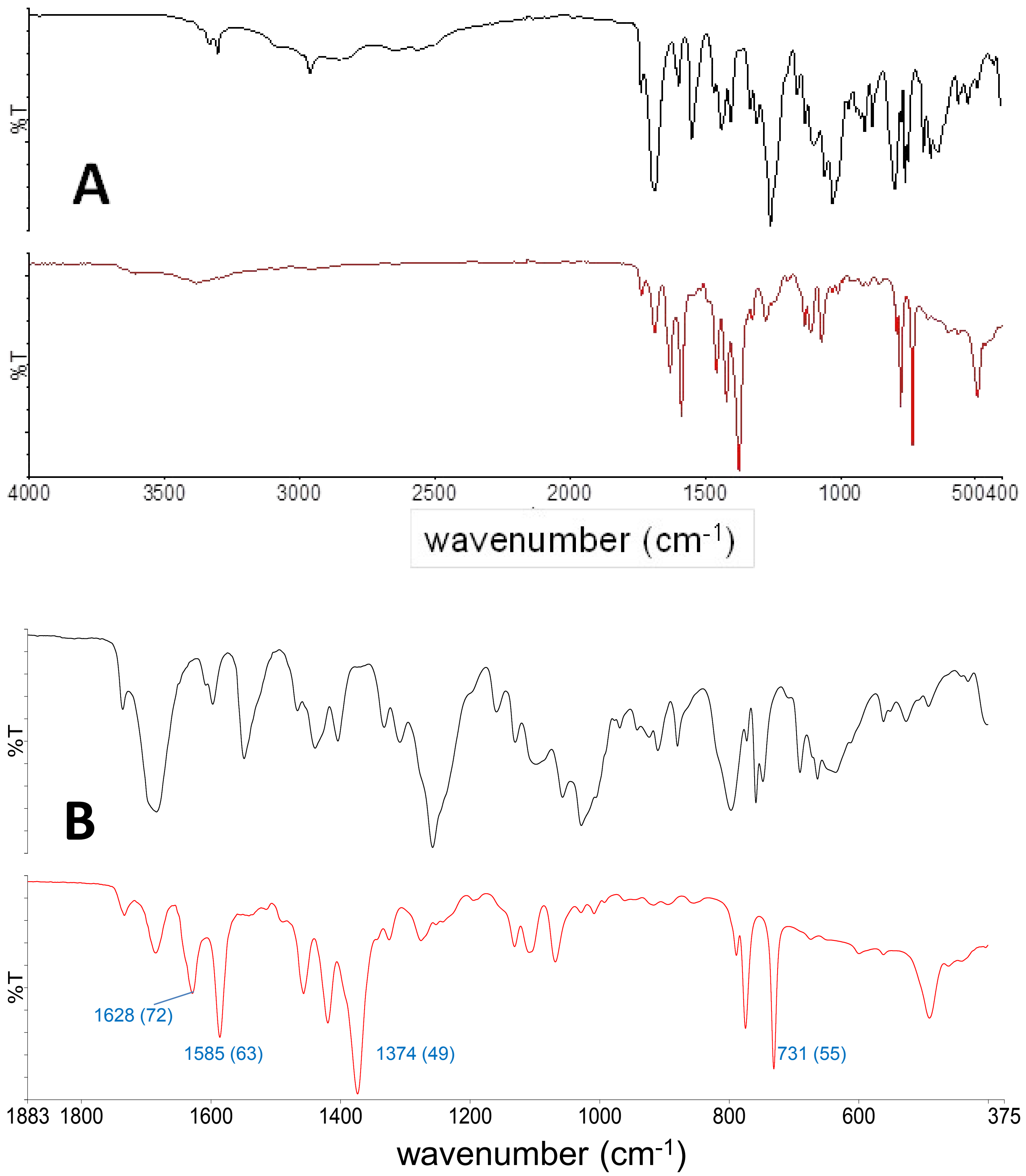

Figure S13. (**A**) ATR-FTIR spectra of 1,3-H_2YBDC (black curve) and [Cu(1,3-YBDC)]·xH_2O (red curve); (**B**) magnification of the 1800-400 cm^{-1} range (in parentheses the % Transmittance values of the labelled peaks).

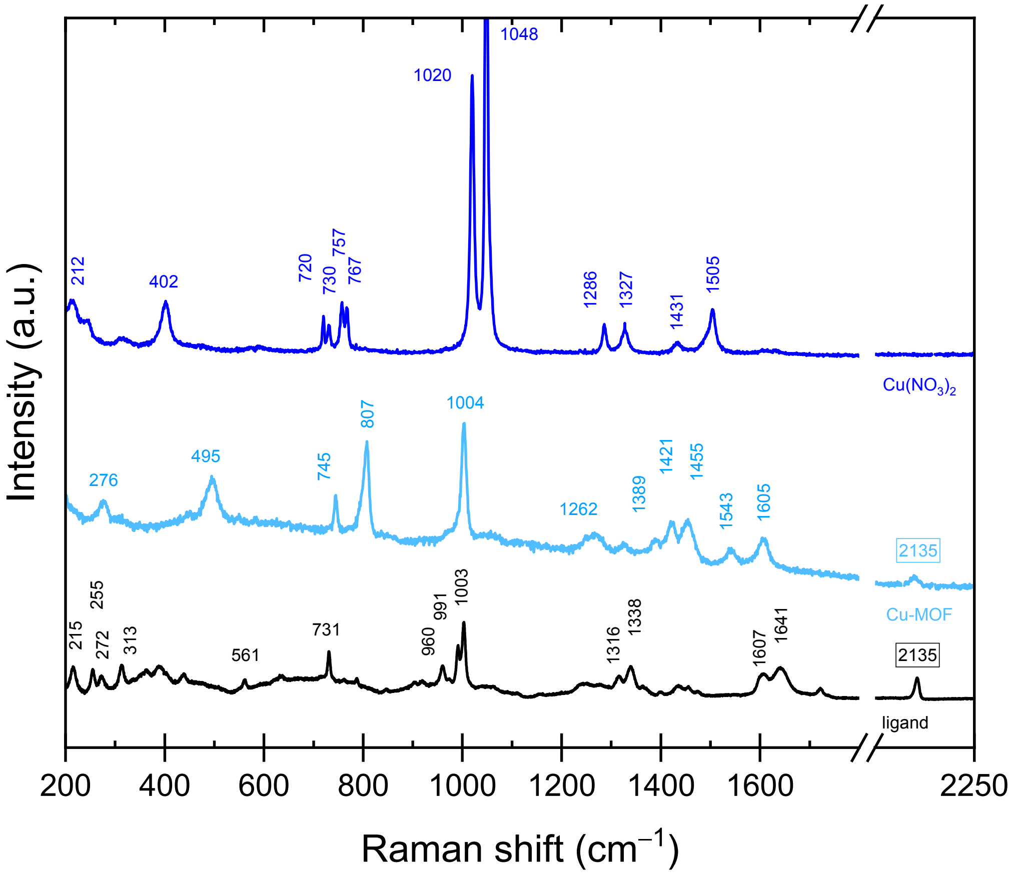

Figure S14. Raman spectra of 1,3-H_2YBDC, $Cu(NO_3)_2 \cdot 2.5H_2O$ and [Cu(1,3-YBDC)]·xH_2O.

S.2.2. X-ray diffraction investigation

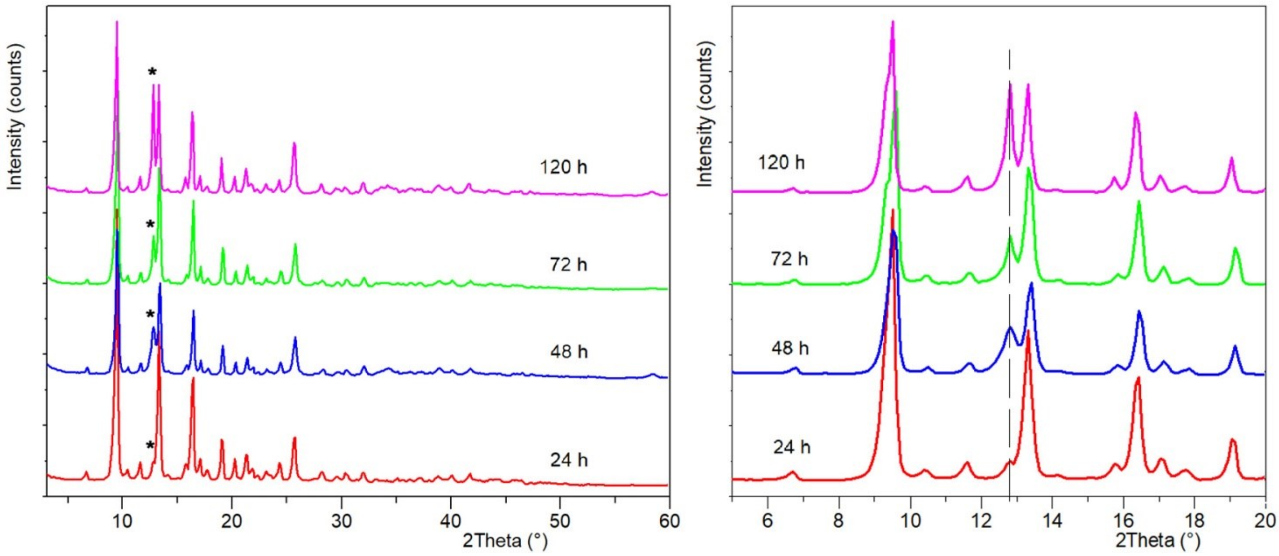

Figure S15. **Left**: full plot of diffraction patterns collected on a Panalytical X'Pert Pro (Cu Kα radiation, λ = 0.154 nm, 40 mA, 40 kV) diffractometer in Bragg-Brentano mode for samples reacted in 2-propanol at different reaction times (24, 48, 72 and 120 h). **Right:** enlargement between 5 and 20° 2θ. The starred peaks as 12.82° (vertical dashed line in the enlargement) corresponds to the (001) refection of the highly textured $Cu_2(OH)_3(NO_3)$ contaminant species.

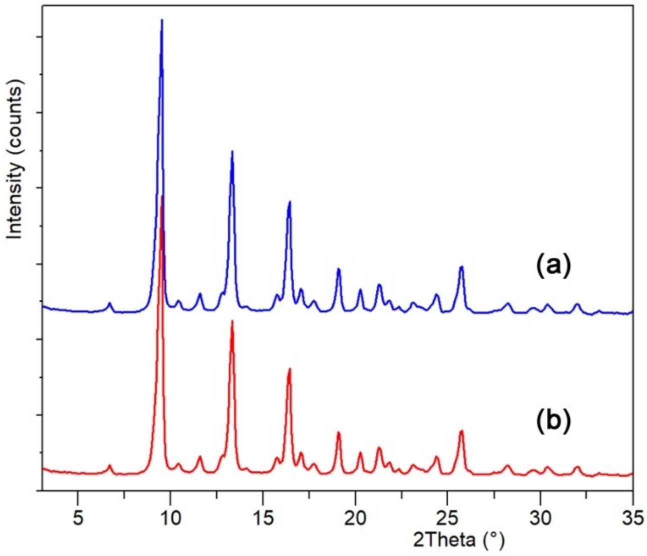

Figure S16. Diffraction patterns of [Cu(1,3-YBDC)]·$x$$H_2O$, collected (a) before and (b) after suspending it in water for 24h.

S.2.3. Atomic absorption spectroscopy (AAS). The overall amount of copper present on the different samples was determined by means of flame atomic absorption spectroscopy (AAS, Thermo Scientific iCE 3300 AA01124707) in air-acetylene flame (λ = 324.8 nm; spectral bandwidth = 0.5 nm). The analyses were conducted by comparison with five calibration standards (2.0, 4.0, 6.0, 8.0, 10.0 ppm) prepared by dilution to 25 mL of different amounts of a 100 ppm standard solution prepared by diluting 1 mL of *FIXANAL* (03372-1EA Fluka, Copper atomic spectroscopy standard concentrate 10.00 g/L*)* in 0.5 M HNO_3 (Normatom®, 67-69 %, d = 1.41 g/cm^3, MW 63.01, VWR Chemicals). The samples were prepared by first heating the solid (ca. 5 mg) with concentrated nitric acid until complete dissolution and subsequently diluted with HNO_3 0.5 M up to a volume of 100 mL.

S.2.4 Ab-initio crystal structure solution from synchrotron X-ray diffraction data. Unit cell parameters determination was carried out by the standard peak search methods, followed by profile fitting for the accurate estimate of the peak positions in the 2°-7° 2θ range, which, through the SVD indexing algorithm[3] implemented in TOPAS-R,[4] provided a primitive tetragonal cell [a = b = 18.190 Å, c = 18.872 Å, GOF(20) = 212.2]. Space group determination through the analysis of systematic absences indicated *P4/ncc*, later confirmed by successful structure solution/refinement.

Structure solution was performed by Monte Carlo/Simulated Annealing technique using a freely floating Cu atoms and a rigid model, flexible at all saturated torsional angles for the organic molecule described by the Z-matrix formalism with idealized geometrical parameters. Once a plausible solution was found, the analysis of the structural model provided further hints toward the presence of a structurally disordered chain (with two alternative conformations of estimated 0.51(1):0.49(1) site occupancy factors) and of extra residual electron density in the crystal cavities, interpreted by positioning two light atoms (here, oxygen), as if water molecules were hosted in the MOF channels. For simplicity, and fully aware that in this kind of channel hydrates the water content may vary depending on the environmental conditions,[5] such as temperature and relative humidity, we have adopted for this (nearly dihydrate) solid phase the nominal stoichiometric

formula [Cu(1,3-YBDC)]·$2H_2O$. The final refinements were carried out by the Rietveld method, maintaining the rigid bodies introduced at the structure solution stage. The background was modelled by a polynomial function of the Chebyshev type, peak profiles were described by the Fundamental Parameters Approach;[6] two isotropic thermal factors were refined, one attributed to all framework and one to the propargyl-carbamate branch atoms, respectively. In spite of the transmission geometry of the instrument and the capillary spinning during data acquisition, minor texture effects were present. A preferred orientation correction was therefore applied in the form of a three-parameters spherical harmonics description.[7] Further analysis of the experimental pattern suggested that contaminant nanocrystalline phases were also present, and attributed to $Cu_2(OH)_3(NO_3)$ (monoclinic $P2_1$, average crystal size = 25 nm)[8] and $Cu(OH)_2$ (orthorhombic $Cmc2_1$, mean crystal size = 11 nm).[9] Due to a large texturing effect, their contribution was modelled by adding a structureless description (in the LeBail mode)[10] in the final refinement cycles. The final Rietveld refinement plot is shown in Figure S17.

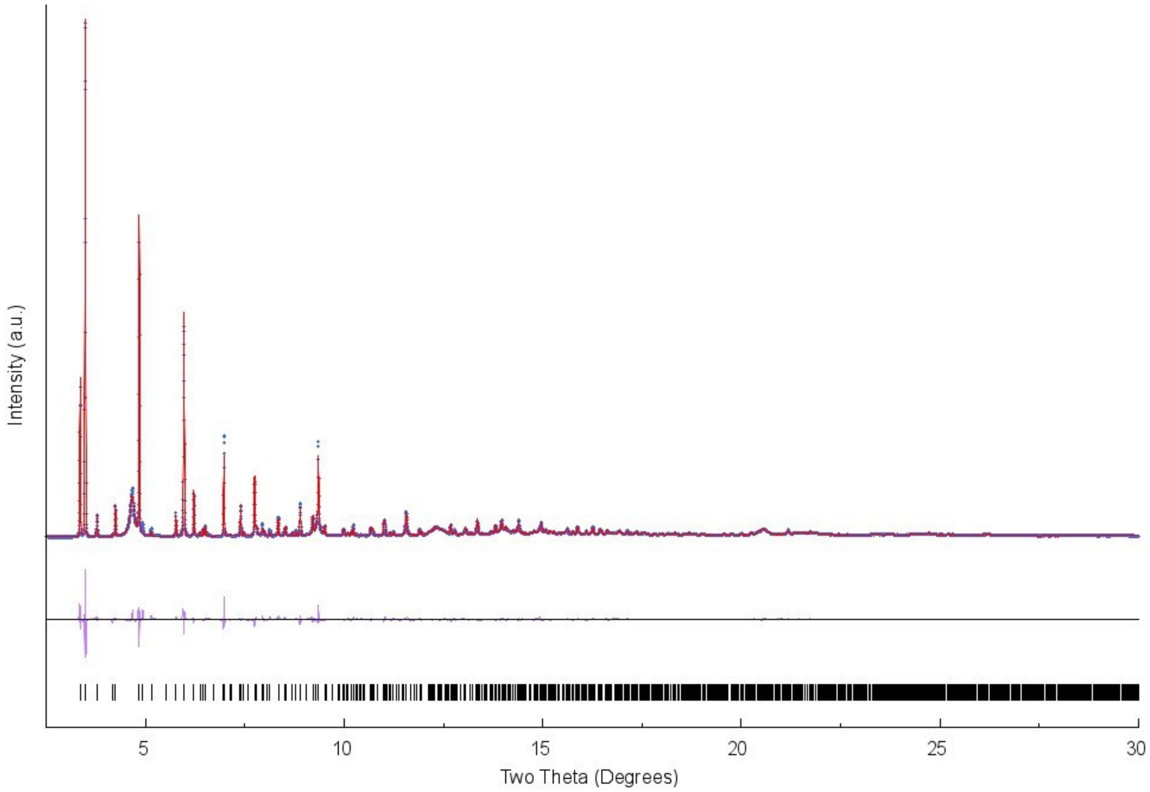

Figure S17. Rietveld refinement plot for [Cu(1,3-YBDC)]·$2H_2O$; Blue trace = observed data; red trace = calculated pattern. Difference plot in purple; peak markers at the bottom are for [Cu(1,3-YBDC)]·$2H_2O$.

Supplementary References

1 E. Marouseau, A. Neckebroeck, H. Larkin, A. Le Roux, L. Volkov, C. L. Lavoie and É. Marsault, *RSC Adv.*, 2017, **7**, 6059–6063.

2 D. L. Boger, P. Ducray, W. Chai, W. Jiang and J. Goldberg, *Bioorganic Med. Chem. Lett.*, 1998, **8**, 2339–2344.

3 A. A. Coelho, *J. Appl. Crystallogr.*, 2003, **36**, 86–95.

4 TOPAS R. Bruker AXS: Karlsruhe, Germany

5 S. Byrn, G. Zografi and X. Chen, in *Solid State Properties of Pharmaceutical Materials*, John Wiley & Sons, 2017, pp. 38–47.

6 R. W. Cheary and A. Coelho, *J. Appl. Cryst.*, 1992, **25**, 109–121.

7 M. Ahtee, M. Nurmela, P. Suortti and M. Järvinen, *J. Appl. Cryst.*, 1989, **22**, 261–268.

8 H. Effenberger, *Zeitschrift fur Krist. - New Cryst. Struct.*, 1983, **165**, 127–135.

9 H. R. Oswald, A. Reller, H. W. Dubler and E. Schmalle, *Acta Crystallogr. Sect. C Cryst. Struct. Commun.*, 1990, **46**, 2279–2284.

10 A. Le Bail, *Powder Diffr.*, 2005, **20**, 316–326.
